# Hierarchical Aggregation in a Complex Fluid—The Role of Isomeric Interconversion

**DOI:** 10.1021/acs.jpcb.2c07527

**Published:** 2023-02-23

**Authors:** Daniel Massey, Christopher D. Williams, Junju Mu, Andrew J. Masters, Ryuhei Motokawa, Noboru Aoyagi, Yuki Ueda, Mark R. Antonio

**Affiliations:** † Department of Chemical Engineering, School of Engineering, The University of Manchester, Oxford Road, Manchester M13 9PL, United Kingdom; ‡ Dalian Institute of Chemical Physics, CAS, 457 Zhongshan Road, Dalian 116023, China; § Materials Sciences Research Center, Japan Atomic Energy Agency, Tokai, Ibaraki 319-1195, Japan; ∥ Advanced Science Research Center, Japan Atomic Energy Agency, Tokai, Ibaraki 319-1195, Japan; ⊥ Department of Chemistry, Colorado School of Mines, Golden, Colorado 80401, United States

## Abstract

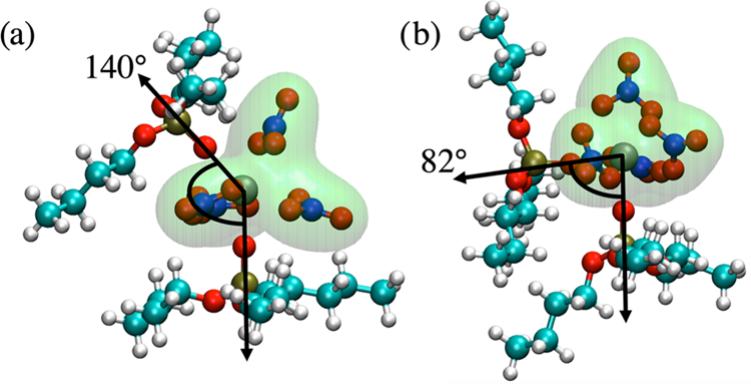

There is an ever-increasing body of evidence that metallic complexes involving amphiliphic ligands do not form normal solutions in organic solvents. Instead, they form complex fluids with intricate structures. For example, the metallic complexes may aggregate into clusters, and these clusters themselves may aggregate into superclusters. To gain a deeper insight into the mechanisms at play, we have used an improved force field to conduct extensive molecular dynamics simulations of a system composed of zirconium nitrate, water, nitric acid, tri-*n*-butyl phosphate, and *n*-octane. The important new finding is that a dynamic equilibrium between the cis and trans isomers of the metal complex is likely to play a key role in the aggregation behavior. The isolated cis and trans isomers have similar energies, but simulation indicates that the clusters consist predominantly of cis isomers. With increasing metal concentration, we hypothesize that more clustering occurs and the chemical equilibrium shifts toward the cis isomer. It is possible that such isomeric effects play a role in the liquid–liquid extraction of other species and the inclusion of such effects in flow sheet modeling may lead to a better description of the process.

## Introduction

The most common way to separate metals is liquid–liquid extraction.^1−8^ Taking nuclear fuel recycling as an important example, spent nuclear fuel is delay-stored to reduce the effects of short-lived radioactive species, and then this material is dissolved in nitric acid. The acid solution, denoted as the aqueous phase, is contacted with an organic diluent and an appropriate ligand. The ligand binds selectively to the desired metal to form a complex, and this complex moves from the aqueous to the organic phase, thus achieving the separation. An important example of this is the plutonium uranium reduction extraction (PUREX) process for extracting uranium and plutonium. This was patented in 1947 and is still in common use.^9,10^ The organic phase containing the extracted metal is far from a simple solution, however. Both atomistic computer simulations, small-angle neutron (SANS) and X-ray (SAXS) scattering experiments indicate the presence of discrete aggregated structures resulting from a complex interplay between the interactions between metal complexes and the fluid structures resulting from having an amphiphilic ligand in a nonpolar environment.^1,7,11−20^ An interesting system that illustrates this complexity is that of zirconium nitrate, water, nitric acid, tri-*n*-butyl phosphate (TBP), and *n*-octane.^21^ Extended X-ray adsorption fine structure (EXAFS) studies indicated that zirconium formed the neutral complex Zr(NO_3_)_4_(TBP)_2_. A careful analysis of the neutron scattering profiles indicated a hierarchical aggregation of these complexes, first into primary clusters and then into superclusters. At the highest zirconium concentration studied, just under the critical concentration where there is a transition to the third phase,^22,23^ there were approximately seven complexes in a primary cluster and 25 primary clusters in a supercluster. Molecular dynamics (MD) simulations, using the trans stereoisomer of the complex, also showed aggregation of the complexes into primary clusters and were able to provide insight into the detailed molecular arrangements within these clusters. The force field parameters used, however, resulted in unphysical interatomic distances between the zirconium and ligand atoms. After improving the force field, our simulations no longer showed the same degree of clustering observed in our previous simulations.

In this article, we revisit this problem. We present evidence that there is a dynamical chemical equilibrium between the trans and cis stereoisomers of the complex and that it is the cis isomer that is predominantly involved in the aggregation of the complexes. The structure of the article is as follows. We first present details of density functional calculations on the structure and energetics of the two stereoisomers and describe the revised force field used for the simulations. We then report simulation results on the properties of an all-cis system, an all-trans system, and a cis/trans mixture. We go on to present the potential of mean force (PMF) simulations for the formation of dimers of the complexes. We use these results to gain insights into the molecular driving forces for aggregation. We then present an isodesmic aggregation model, yielding predictions for the pure cis and pure trans systems in reasonable agreement with simulation. We finish off with a discussion.

## Results and Discussion

### Quantum Mechanical Calculations

Density functional theory (DFT) calculations were employed to assess the relative stabilities of various stereoisomers of the Zr(NO_3_)_4_(TBP)_2_ complex. Starting with atomic coordinates obtained from our previous study,^21^ the geometry of the complex was re-optimized using the B3LYP hybrid functional,^24,25^ the LanL2DZ effective core potential basis set for Zr,^26^ and the 6-311++G(3df,2p) basis set^27−29^ for all other atom types. In addition, a conformational energy search was performed to find the lowest energy stereoisomers; the positions of the various NO_3_^–^ and TBP ligands were exchanged and, separately, the TBP ligands separately rotated about the Zr–O=P axis, followed by re-optimization. All geometry optimizations were carried out using the conductor-like polarizable continuum model (CPCM)^30−33^ to implicitly mimic the effect of solvation by *n*-octane (dielectric constant = 1.92). The Gaussian 09 package was used for all DFT calculations.^34^

**Figure 1:**
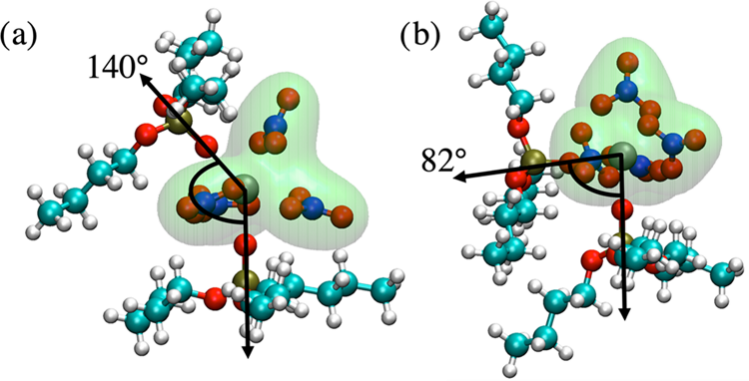
Structures of the lowest energy (a) trans isomer and (b) cis isomer, where Zr, P, O, N, C, and H atoms are represented with gray, gold, red, blue, cyan, and white spheres, respectively. A molecular surface over all nitrate atoms is shown in green to highlight the accessibility of the polar patch.

The resulting optimized stereoisomers can be broadly categorized according to their (TBP)O–Zr–O(TBP) angle as cis (∼80°) or trans (∼140°). Here, (TBP)O refers to the phosphoryl oxygen atom that is directly bonded to the Zr. Although the energies of the various isomers differed by as much as 10 kJ mol^–1^, the lowest energy cis isomer was only 5.8 kJ mol^–1^ higher in energy than the lowest energy trans isomer. Therefore, neither the presence of the cis isomer in the complex fluid nor its participation in cluster formation can be ruled out, especially considering the errors associated with the DFT energy calculation, in particular those arising from the use of an implicit solvent model. Figure 1 shows the structures of the two lowest energy cis and trans isomers. Inspection of the structures shows that while the trans isomer had a rather complete hydrocarbon coating surrounding the Zr atom, the cis isomer had an exposed polar patch. The dipole moments of the two species were 6.4528 D (trans) and 15.2239 D (cis), consistent with this polar patch picture. It should be noted that the calculated bond distances are very similar for the trans and cis stereoisomers. Furthermore, both sets are in good agreement with the average bond distances evaluated by Zr K-edge EXAFS.^21^ Unfortunately, however, this lack of variation means that current experimental data cannot tell us which stereoisomer is most prevalent.

The atomic coordinates of the two lowest energy isomers were used as input structures for the Zr(NO_3_)_4_(TBP)_2_ complex in the molecular dynamics (MD) simulations. The Merz–Singh–Kollman scheme^35,36^ was employed to obtain the partial atomic charges for the two complexes by fitting to the molecular electrostatic potentials, and these charges were employed in the MD simulations. The atomic coordinates and partial charges for these complexes are provided in Section S1 of the Supporting Information.

As the focus of this article is the association of these Zr complexes into clusters, we used DFT to optimize a trans dimer structure to see whether dimerization significantly affected the geometry of the individual Zr complexes. To do this, we first ran a potential of mean force (PMF) simulation, where we pulled two complexes together in explicit solvent until we reached the position of the PMF minimum. Full details are given in a later section of this article. We used the final configuration of the two complexes, as generated in this molecular dynamics simulation, as the starting point for geometry optimization. We did this in vacuo using the B3LYP hybrid functional and 3-21G basis set. While there were changes in bond lengths and bond angles, these were relatively small, and there was no sign of any ligand bridging the two complexes. The two complexes remained intact. Naturally, this is not a definitive result, as that would require an investigation of basis set dependence, a scan of many possible initial configurations, a study of solvent effects, and a study of the various complex isomers, but we take it as a strong indication that we may proceed with simulations on the assumption that the complexes do not significantly change structure upon forming a cluster.

### Simulation Details

The Gromacs package^37−44^ was used for all MD simulations. We used cubic periodic boundary conditions, and the leapfrog algorithm^45^ was used to integrate the equations of motion with a time step of 1 fs. Short-range Lennard-Jones parameters were modeled using the Optimized Potentials for Liquid Simulations (OPLS) force field; parameters are provided in full in our previous publications, and the quality of the force field is discussed.^16,21^ Zr–ligand distances and ligand–Zr–ligand angles were maintained at their equilibrium distances and angles using harmonic potentials with force constants of 2.184 × 10^5^ kJ mol^–1^ nm^–2^ and 1.255 × 10^3^ kJ mol^–1^ rad^–2^. Short-range interactions were taken to zero between 0.9 and 1.2 nm using a switch function. For the long-range electrostatic potential, the particle mesh Ewald method was applied, using a real space cutoff of 1.2 nm.^46^ All simulations were conducted in the isothermal–isobaric (*NpT*) ensemble, where the pressure and temperature were set to be 1 bar and 298 K, respectively. For each system considered, equilibration runs were performed using the velocity-rescaling thermostat^47^ and the Berendsen barostat.^48^ A time constant of 1 ps was used for the Berendsen barostat. In some cases, the initial configuration was minimized prior to equilibration using the steepest descent algorithm. The Nosé–Hoover thermostat^49,50^ and Parrinello–Rahman barostat^51,52^ were applied in subsequent production runs, during which the simulation results were generated. The time constants used for the Nosé–Hoover thermostat and Parrinello–Rahman barostat were 0.2 and 5.0 ps, respectively. In all constant pressure simulations, isotropic pressure coupling was employed using a compressibility of 4.5 × 10^–5^ bar^–1^. All chemical bonds involving hydrogen atoms were constrained using LINCS with a restraining force of 1000 kJ mol^–1^ nm^–2^.

### Clustering Simulations

To investigate possible clustering, we conducted MD simulations on systems containing Zr(NO_3_)_4_(TBP)_2_ complexes, water, nitric acid, TBP, and *n*-octane. We considered a composition corresponding to System 5 in the work by Motokawa et al.,^21^ which had the highest concentration of zirconium studied. The volume fractions of zirconium complex, nitric acid, water, TBP, and *n*-octane in the organic phase were 0.019, 0.011, 0.001, 0.127, and 0.845, respectively. In each simulation, a total of 40 zirconium complexes were considered in the presence of 548 molecules of TBP, 392 molecules of HNO_3_, 70 molecules of H_2_O, and 6522 molecules of *n*-octane, the organic diluent. This closely reproduces the composition of the experimental system given above. We simulated pure trans isomers, pure cis isomers, and a 50:50 mix of cis and trans. Each system was equilibrated for 20 ns, followed by an 80 ns production run. Our force field does not allow interconversion between isomers. The force field does not permit a ligand to leave a complex, and there is not enough space for a new ligand to attach. Steric hindrance prevents the rearrangement of the ligands around the Zr atom. We checked this point by examining the output of our simulations. The densities of these three systems were 0.7026 g cm^–3^ (cis), 0.7053 g cm^–3^ (trans), and 0.7036 g cm^–3^ (mixed), while the experimental density was 0.7568 g cm^–3^. Clearly, there is room for improvement in the force field but, as discussed previously, the challenge with constructing a nonpolarizable force field to deal with systems such as this is that one needs to find parameters for ligands, such as TBP, which adequately treat interactions with both polar and nonpolar molecules. We have endeavored to find a good compromise in our previous studies,^14,21,53^ but while for metal-free systems, this permits reasonable agreement with SANS and SAXS scattering profiles, the density predictions are somewhat compromised. It is also important to note that the density of the solvent, *n*-octane, is slightly under-predicted by the OPLS force field, *viz*., 0.685 g cm^–3^ as opposed to the experimental value of 0.703 g cm^–3^.^54^ We are hopeful that the difference in densities is small enough to show our force field is reasonable, especially as our aim here is to uncover basic trends rather than to make precise predictions.

**Figure 2:**
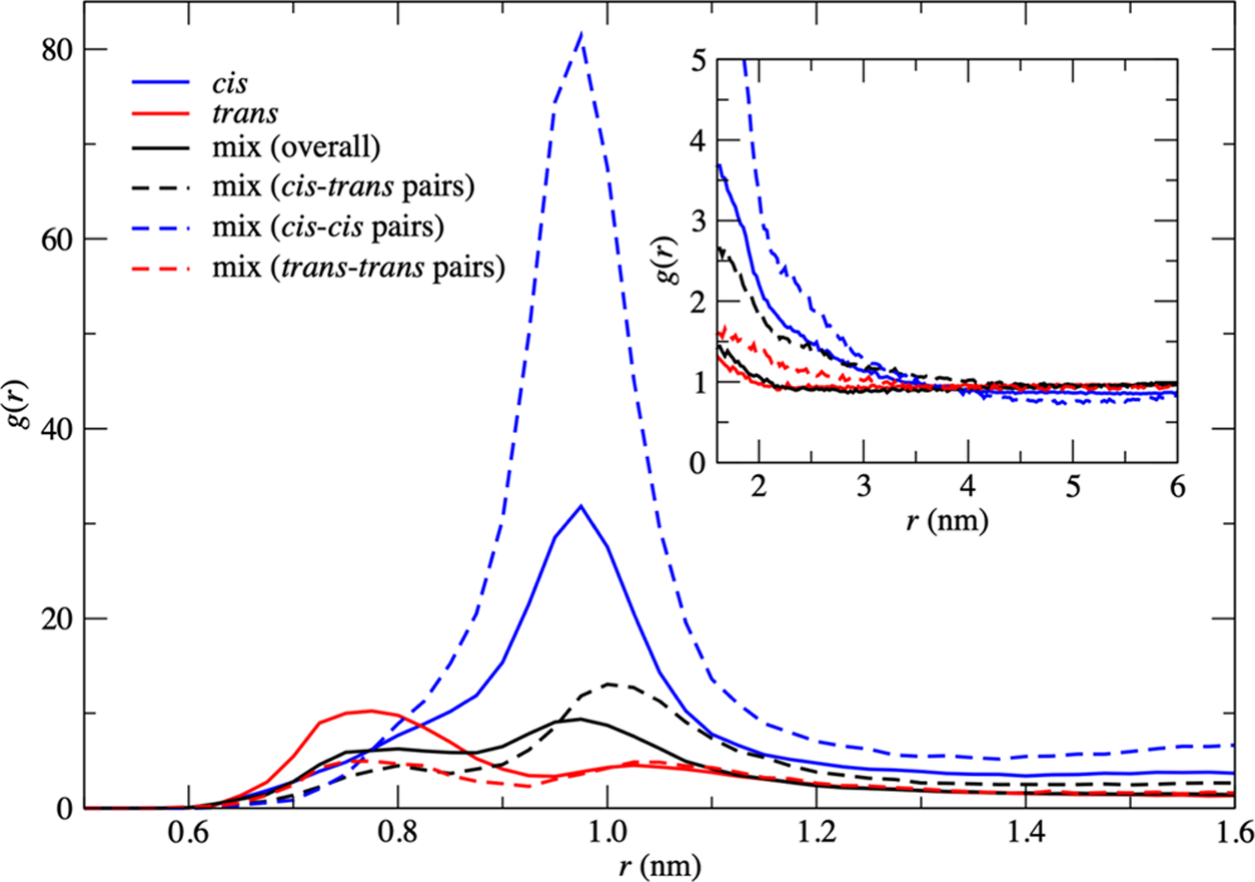
*g*(*r*_Zr–Zr_) obtained from pure cis (blue, solid line), pure trans (red, solid line), and mixed cis–trans systems (black line). Separate plots of *g*(*r*_Zr–Zr_) for cis–cis, trans–trans, and cis–trans pairs obtained from the cis/trans mixture simulation are shown with dashed blue, red, and black lines, respectively. The inset figure shows that the radial distribution functions tend to one at distances greater than ∼4 nm.

To analyze this clustering quantitatively, we used the algorithm of Sevick,^55^ with the criterion that two Zr complexes were connected if the Zr–Zr distance was less than a predefined cutoff. The cutoff was chosen to be 1.25 nm, which is the approximate distance at which the first peak in the Zr–Zr radial distribution function, *g*(*r*_Zr–Zr_), in the cis system ends (Figure 2). The choice of cutoff is somewhat arbitrary, but the resulting analysis is in reasonable agreement with the conclusions drawn from observing many snapshots. For the sake of clarity, we consider a monomer to be a cluster with an aggregation number of one for the purposes of this analysis. The cluster size distribution, mean cluster size, maximum cluster size, and the number of clusters were monitored throughout the production simulation. Section S2 of the Supporting Information presents data on the sensitivity of the cluster distributions to the distance criterion. *g*(*r*_Zr–Zr_) was also calculated for the trans, cis, and mixed systems, and the corresponding coordination numbers CN(*r*_Zr–Zr_) were also calculated. In addition, radial distribution functions were calculated for Zr–ligand pairs to elucidate the solvation environment of clustered and isolated complexes as well as to investigate possible intermediary roles played by other species.

The *g*(*r*_Zr–Zr_) functions shown in Figure 2 consist of a large peak followed by an extended tail. This large peak, which corresponds to the closest Zr–Zr distance on average, reaches its maximum at approximately 0.99 and 0.79 nm for the pure cis and trans systems, respectively. The pure trans *g*(*r*_Zr–Zr_) also has a shoulder at 1.06 nm, suggesting the possibility of two separate clustering conformations. Two distinct peaks are visible in *g*(*r*_Zr–Zr_) for the mixed system at 0.80 and 0.97 nm, the latter of which is more intense. Since these peak positions are similar to those of the pure simulations, this suggests the presence of cis–cis and trans–trans complexes in the mix. The radial distribution function for the mix can be decomposed into separate contributions due to cis–cis, trans–trans, and cis–trans pairs, which are shown with dashed lines in Figure 2. In the mixed cis/trans system, the peak intensity for cis–cis pairs is greatly increased, demonstrating that a greater proportion of cis complexes are involved in clustering than in the pure analogue. In contrast, the peak intensity for trans–trans pairs is greatly reduced compared to the pure system, demonstrating that a smaller proportion of trans complexes are involved in clustering than in the mixed system. Interestingly, the position of the primary peak in the mixed system is closer to the *R*_S_ value of 0.92 ± 0.03 nm in the characteristic parameters of the hierarchical aggregates model^21^ than either of the peak positions obtained from the pure cis or pure trans simulations.

The fact that these radial distribution functions exhibit more structure than a single peak is interesting, and we will return to this matter later.

**Figure 3:**
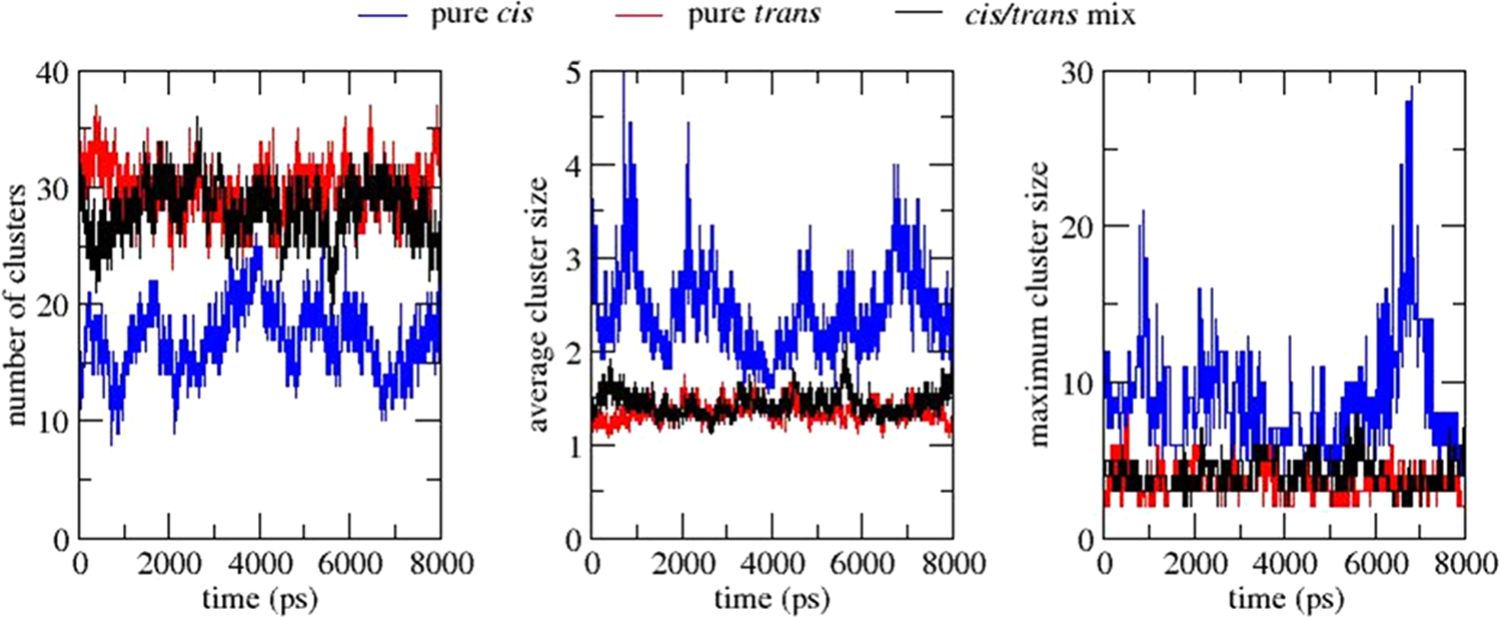
Number of clusters (left), average cluster size (center), and maximum cluster size (right) for the pure cis (blue), pure trans (red), and cis/trans mix (black) systems with respect to simulation time.

For each of the three systems, i.e., pure cis, pure trans, and the cis/trans mixture, the time evolutions of the number of clusters, average cluster size, and maximum cluster size are plotted in Figure 3. Throughout the simulation trajectory, the number of clusters in the pure trans and mixed systems is ∼50% higher than that of the pure cis system. The average cluster size of the pure trans and mixed systems is in the range of 1–2 complexes, in contrast to the pure cis system, which is consistently greater than 2 complexes, with occasional spikes above 3 complexes. The maximum cluster size in the pure trans and mixed systems rarely increases beyond 5 complexes, whereas in the cis system, larger clusters are frequently observed. In the latter case, the maximum cluster size occasionally sees large spikes (e.g., at 1000 and 7000 ps) before returning to the normal range. The above observations, combined with visual analysis, demonstrate that clustering is dynamic, with frequent association and dissociation events occurring throughout the simulation.

**Figure 4:**
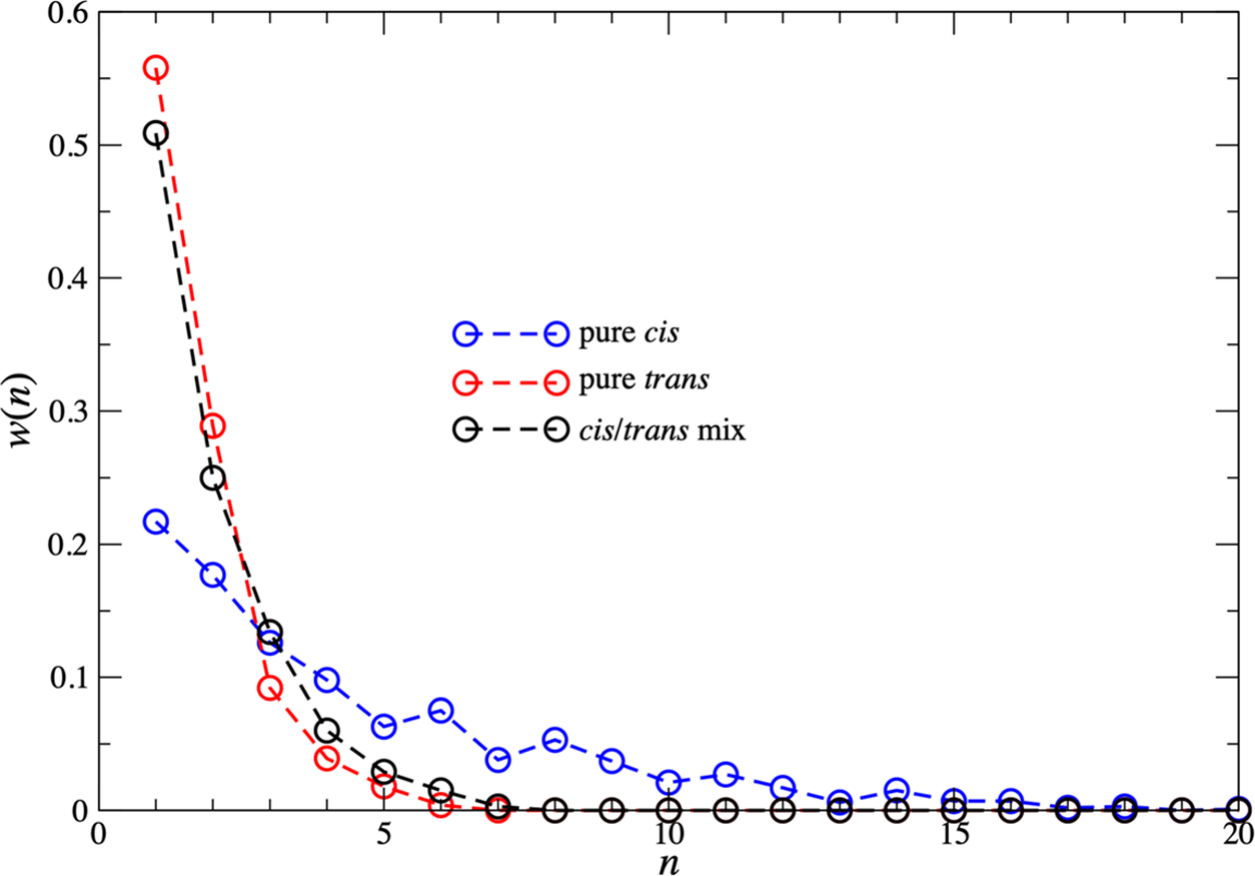
Size-weighted cluster size probability distribution, *w*(*n*), for the pure cis (blue), pure trans (red), and cis/trans mix (black) systems. Here, *w*(*n*) is the fraction of complexes contained in aggregates of *n* complexes.

**Figure 5:**
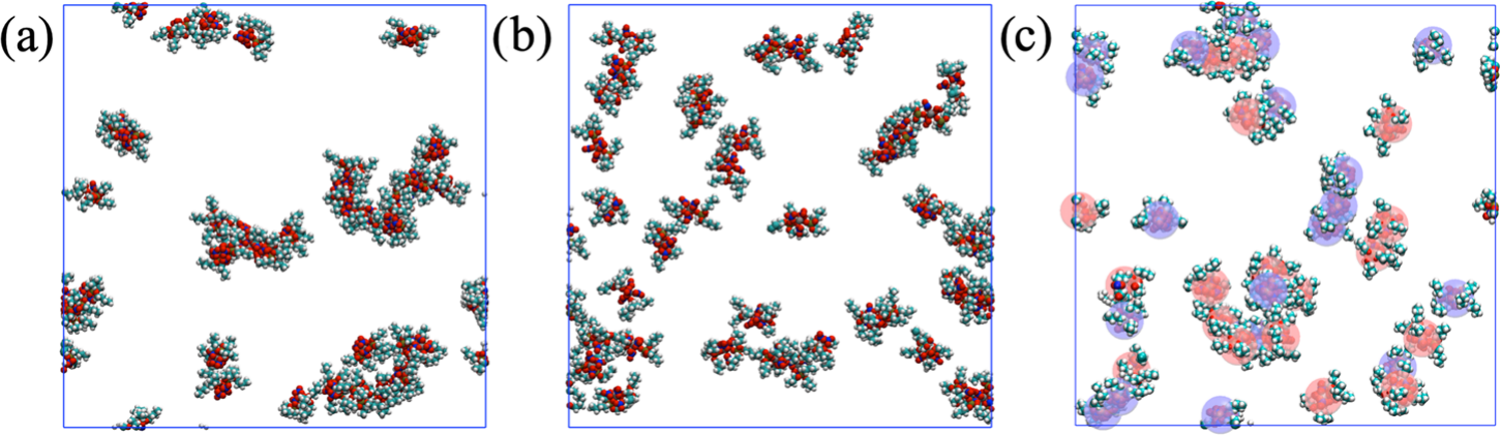
Snapshots of the pure cis ((a) left), pure trans ((b) center), and cis/trans mix ((c) right) systems. Only atoms contained in the Zr complexes (Zr = gray, P = gold, O = red, N = blue, C = cyan, H = white) are shown for visual clarity. In the cis/trans mixed system, the cis and trans isomers are additionally labeled with blue and red spheres, respectively.

The average size distribution of clusters for each system (Figure 4 and Table 1) shows that isolated complexes and dimers are more prevalent in the pure trans/mixed systems than the cis system, and the number of trimers is approximately equal for all three systems. In contrast, clusters with more than three Zr complexes are much more prevalent in the cis system. Clearly, the clustering strongly depends on the isomeric form of the Zr complex, with the pure cis system displaying a greater degree of clustering and larger aggregates. An additional smaller simulation of the pure cis system was performed, containing only 20 Zr complexes. Figure S1 shows that, although the occasional formation of the very largest clusters (such as those observed in Figure 3) is not possible, the cluster size distribution is not significantly affected by system size effects. Typical simulation snapshots of the three systems are shown in Figure 5.

**Table 1:** Average Cis/Trans Composition for Each Cluster Size in the Cis/Trans Mix

cluster size	trans fraction	cis fraction
1	0.60	0.40
2	0.43	0.57
3	0.38	0.62
4	0.36	0.64
5	0.37	0.63
6	0.31	0.69
7	0.31	0.69
8	0.37	0.65

Analysis of the experimental scattering profile indicated an aggregation number of approximately 7. From simulation, the number/weight aggregation number averages were 2.4/4.5 for pure cis, 1.3/1.7 for pure trans, and 1.4/1.9 for the cis/trans mixture. Because the contribution of an *n*-aggregate to the SANS scattering intensity is proportional to *n*^2^, the weighted average is likely to be the most appropriate for comparison. Further analysis and simulation is needed for a quantitative understanding of why the simulated weight average aggregation number of even the pure cis system is significantly less than that obtained from the analysis of experimental data, but a few comments are in order. The fitting procedure applied to the SANS profile assumed the clusters to be of a single size and to have a spherical geometry, whereas our simulations indicate a size and shape distribution of clusters. It is not clear how taking account of this shape and size polydispersity would affect the fitting analysis. Thus, “7” should not be regarded as a particularly stable aggregation number but more as a reasonable estimate of an average cluster size. Naturally, there are caveats on the simulation front also, for example, system size and the quality of the force field.

It would, of course, be useful to calculate neutron scattering profiles from simulation so these may be compared directly with experimental data. We have done these calculations, but the results are uninformative as, unfortunately, our system sizes are too small to probe the low-wave vector regions of the profile that give important information about clustering. We should also note that our system sizes are too small to observe any occurrence of the superclustering reported in the experimental work. Despite all this, however, the fact that the pure cis system shows the most significant degree of aggregation suggests strongly that cis isomers are probably a major component of the experimental system.

An analysis of the lifetime of the clusters shows that trans aggregates are short-lived in comparison to cis aggregates, indicating that the trans isomers are not sticking together to a significant extent. For example, in the cis case, 2111 distinct dimers were observed throughout the course of the simulation. Typically, 77 of these dimers lasted for longer than 1 ns, with the longest-lived dimer remaining intact for 7.7 ns. In contrast, in the trans case, 3657 distinct dimers were observed. Only 32 of these lasted for longer than 1 ns, with the longest-lived dimer remaining intact for just 3.8 ns. For trimers, 22 cis aggregates lasted for longer than 1 ns, with a maximum lifetime of 3.5 ns, but no trans trimer aggregates lasted for longer than 1 ns.

**Figure 6:**
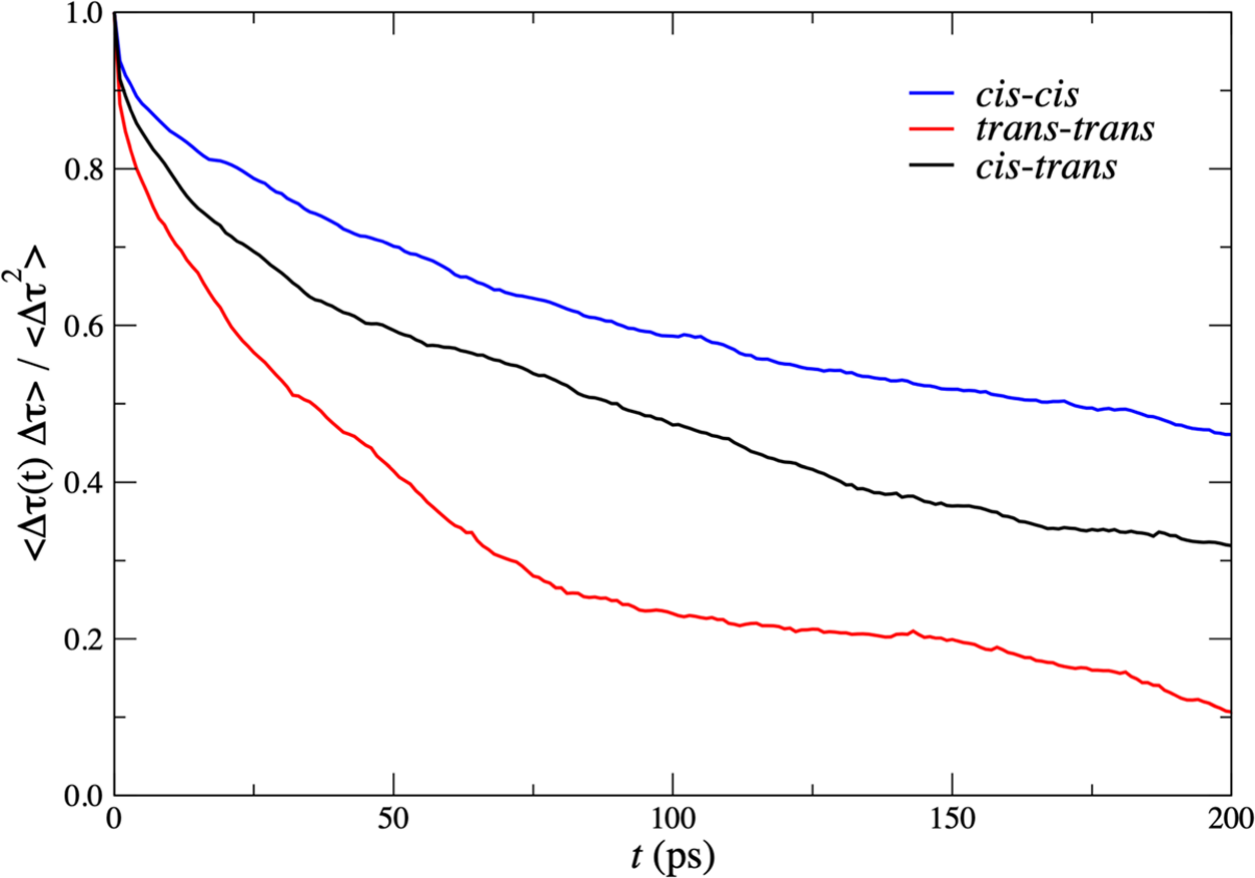
Plot of the normalized time correlation of fluctuations in the mean cluster size, Δτ, for the pure cis (blue), pure trans (red), and the cis/trans mix (black).

We may give a more quantitative account of the differing cluster lifetimes in the three systems by plotting time correlation functions. In Figure 6, we consider the normalized time correlation function of fluctuations of mean cluster size, i.e., the deviation of the mean cluster size at time *t* from the time-averaged cluster size. The half-lives are roughly 35 ps for all-trans, 90 ps for the mixed system, and 170 ps for all-cis.

### Potential of Mean Force Calculations

To gain a deeper thermodynamic understanding of the driving forces for aggregation, we carried out a series of potential of mean force (PMF) calculations. We investigated a system involving only two Zr complexes, where the composition of the water (18 molecules), nitric acid (98 molecules), TBP (137 molecules), and *n*-octane (1631 molecules) corresponded to system 5 in the work by Motokawa et al.^21^ and to the simulations described in the previous section. The PMF calculations were used to determine the Gibbs energy change for the formation of a cis dimer, a trans dimer, and a cis–trans dimer.

Initially, the Zr atoms of the two Zr complexes were fixed within the solvent at a separation of around 3 nm. We first carried out a 10 ns *NpT* equilibration run. We then further equilibrated this system, with the complexes at fixed positions, by carrying out a second *NpT* simulation for 100 ns. This allowed the structures of the complexes to relax and reach equilibrium with the solvent. The final simulation box length was 8.6 nm.

To calculate the PMF, the complexes were pulled toward each other at a pull rate of 0.001 nm ps^–1^ over a period of 2–3 ns. The reaction coordinate was taken to be the distance between the two Zr atoms, and a series of system configurations were collected at reaction coordinate intervals of 0.05 nm. Umbrella sampling (US) simulations were then performed for each interval using a constraining harmonic potential with a spring constant of 1000 kJ mol^–1^ nm^–2^. Again, the equilibration time and production time were 10 and 20 ns, respectively, for each distance. Finally, the weighted histogram analysis method (WHAM) was used to extract the PMF and hence the Gibbs energy of dimerization, Δ_dim_*G.* Bootstrapping was used to obtain error estimates, and these are shown at the minima. These indicate the adequate convergence of the PMFs. We note that because the interaction energies are not greatly in excess of thermal energies, it is not possible to get very smooth curves.

The PMFs obtained in this way do not show plateau regions at large separation but instead exhibit long-range repulsions. This is a well-known, entropically induced artifact and may readily be corrected to yield PMFs that fluctuate about zero at large separations.^56,57^

**Figure 7:**
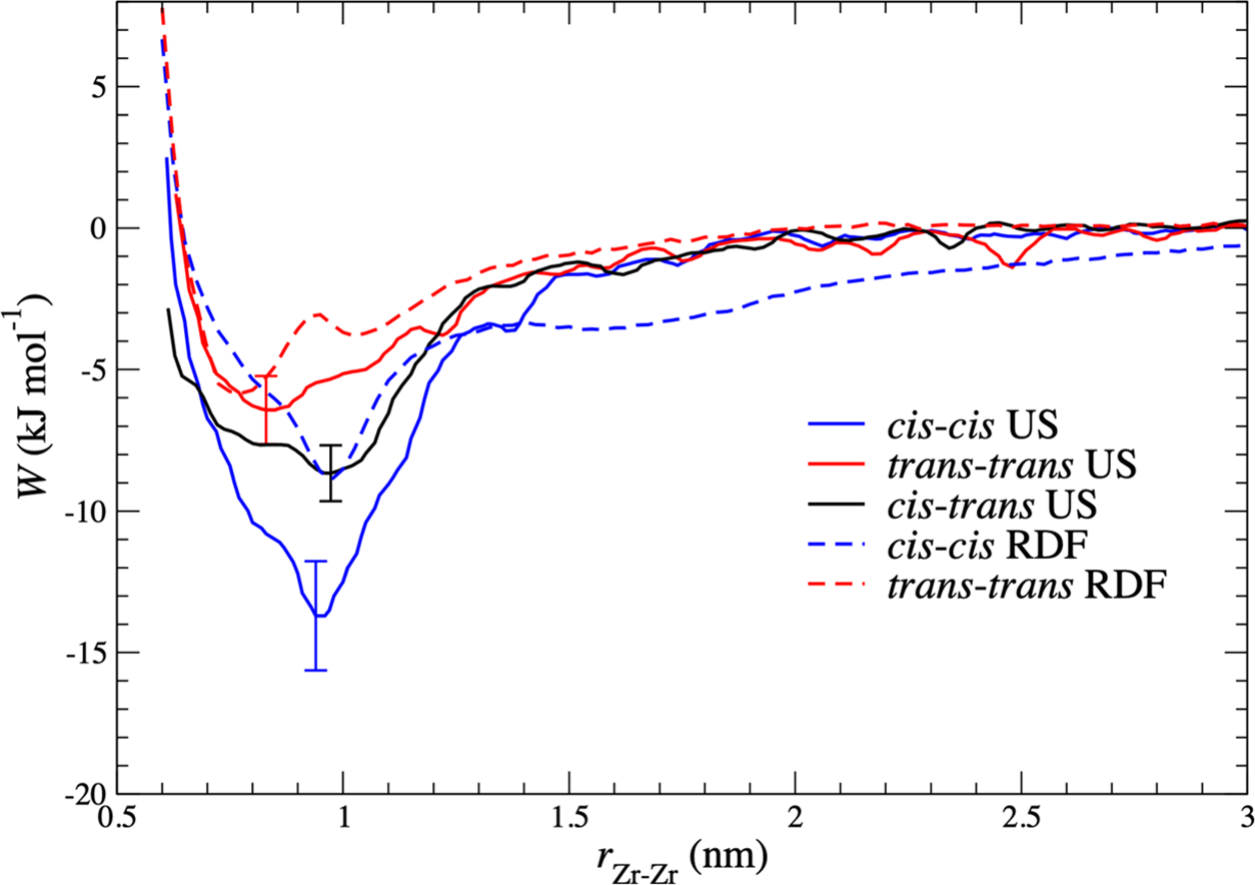
Potentials of mean force (PMFs) for Zr complex aggregation, *W* (kJ mol^–1^), for the pure cis (blue), pure trans (red), and cis/trans mixed (black) systems. The reaction coordinate, *r*_Zr–Zr_, is defined as the distance between the two Zr atoms. PMFs calculated using the umbrella sampling (US) method, shown with solid lines, are compared to estimates from a Boltzmann inversion of *g*(*r*_Zr–Zr_) from the clustering simulations shown in dashed lines.

In Figure 7, the trans–trans, cis–trans, and cis–cis PMFs are plotted. The well depths are 6.4 (±1.2), 8.7 (±1.0), and 13.7 (±1.8) kJ mol^–1^, respectively, in accordance with the observation from simulation that it is the cis isomer that is primarily involved in aggregation.

We now consider the structures of these dimers, focusing on the positions of the minima in the PMF curves. The trans–trans global minimum is at 0.85 nm, while the global minima for cis–trans and cis–cis are at 0.98 and 0.95 nm, respectively. The cis–trans and cis–cis PMFs have shoulders near the position of the trans–trans minimum, while the trans–trans PMF shows a weak feature at the position of the minimum of the cis–trans and cis–cis PMFs. This type of behavior is also apparent in the Zr–Zr radial distribution functions for the bulk simulations, as shown in Figure 2. To show this clearly, we also plot the PMFs obtained from these distribution functions in Figure 7. For the pure cis system, both PMFs show very similar positions for the minimum and also show shoulders at the smaller separations mentioned above. The rdf-derived PMF has a shallower first minimum but a more extended negative region at larger separations. The rdf-PMF also shows a shallow minimum at around 1.6 nm. We attribute the differences to the presence of higher-order clusters. The curves for the pure trans system are similar in magnitude, which we suspect is due to the smaller degree of clustering in comparison with the cis system. The global minimum has shifted to a slightly larger separation in the rdf-derived curve, and the low-density shoulder has transformed to a secondary minimum, again at a slightly larger distance. As Figure 4 shows, there is a degree of clustering in the trans system, which is probably responsible for these changes.

**Figure 8:**
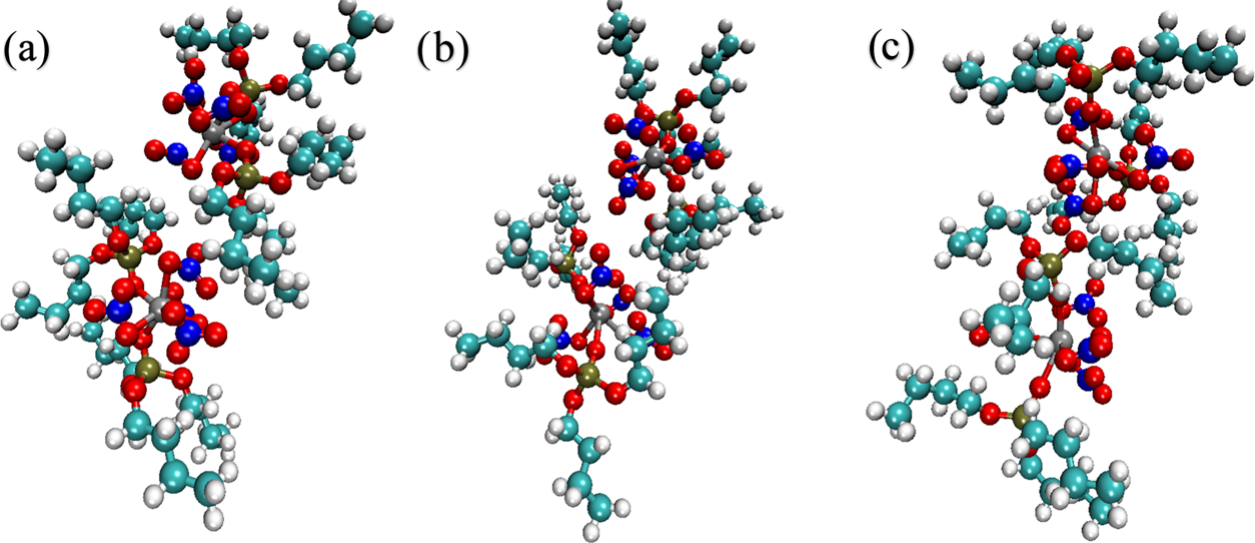
Snapshots of the cis–cis (a), cis–trans (b), and trans–trans (c) dimers at their equilibrium separations, where the Zr, P, O, N, C, and H atoms are represented as gray, gold, red, blue, cyan, and white spheres, respectively.

There would thus appear to be two important distances in the PMFs for all three dimers. Snapshots of the cis–cis, cis–trans, and trans–trans dimers at their equilibrium separations are shown in Figure 8.

To understand the driving forces for the aggregation process, it is helpful to recall that the main difference between a cis and a trans complex is the presence of a polar patch in the cis isomer that is absent for the trans isomer. This is shown in Figure 1. One possible mechanism for aggregation might be the presence of strongly attached bridging molecules, for example, water or nitric acid, which links the nitrate groups. We therefore carried out an extensive analysis of the distribution of water and nitric acid molecules around each complex in the dimer, looking at each type of dimer at Zr–Zr separations of both 0.85 and 0.95 nm. We also calculated these distributions at large separations, where the complexes, to a good approximation, are noninteracting. We present much of this analysis in Section S3 of the Supporting Information, but the upshot is that the distributions and coordination numbers of these species around a complex change only slightly upon dimer formation. A careful study of snapshots indicated no obvious bridging molecules at a 0.95 nm separation, though, at 0.85 nm, there is some evidence of a nitric acid molecule between the two trans complexes. It does not, however, appear to directly bridge the nitrates. We return to the 0.85 nm case later, but the evidence suggests that the driving force for aggregation is unlikely to be connected with strongly attached bridging molecules.

**Figure 9:**
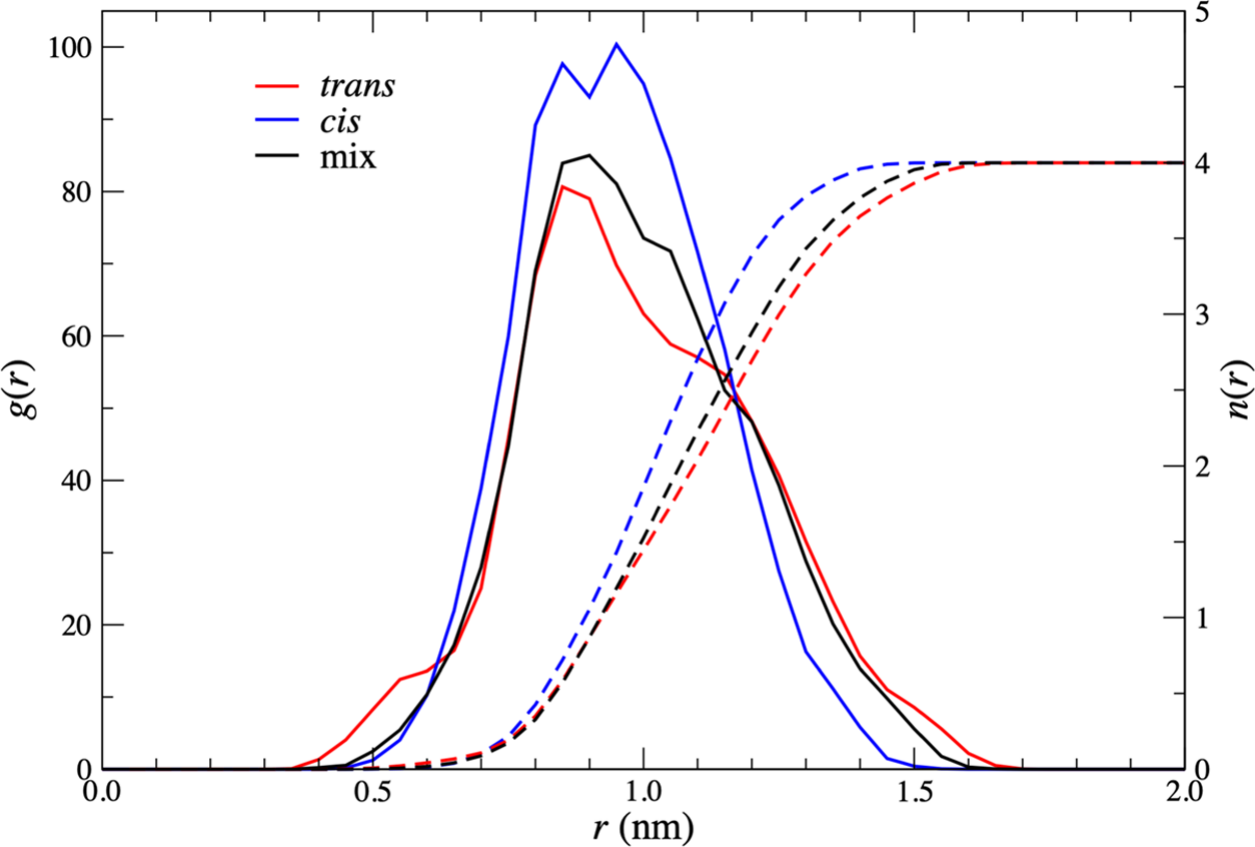
Radial distribution functions, *g*(*r*), (solid lines) and cumulative coordination numbers, *n*(*r*), (dashed lines) for a bound nitrate on one complex, with a bound nitrate on the other. Data are shown for a cis–cis dimer (blue), a cis–trans dimer (black), and a trans–trans dimer (red). The Zr–Zr distance is 0.95 nm, corresponding to the position of the global minimum of the cis–cis PMF.

In Figure 9, we show, for the cis–cis, cis–trans, and trans–trans dimers, the radial distribution functions for a nitrate on one complex, with a nitrate on the other. The distance is that between the nitrogen atoms on each nitrate. Also shown are the cumulative nitrate–nitrate coordination numbers as a function of distance. This corresponds to the average number of nitrates on one complex within a given distance of a single nitrate on the other complex. We have checked that the nitrates are all approximately equivalent, indicating that each complex is free to rotate. At large distances, this coordination number tends to four, as there are four nitrates per complex. These data correspond to a Zr–Zr distance of 0.95 nm, which is the global minimum of the cis–cis PMF. The curves for all three dimers look reasonably similar, showing a strong peak in the radial distribution function in the region of 0.8–0.95 nm. The coordination number plots show that, on average, more than two nitrates are at a greater distance than 0.95 nm away from the central nitrate. This is consistent with a picture that the complexes orient so as to reduce nitrate–nitrate interactions.

**Figure 10:**
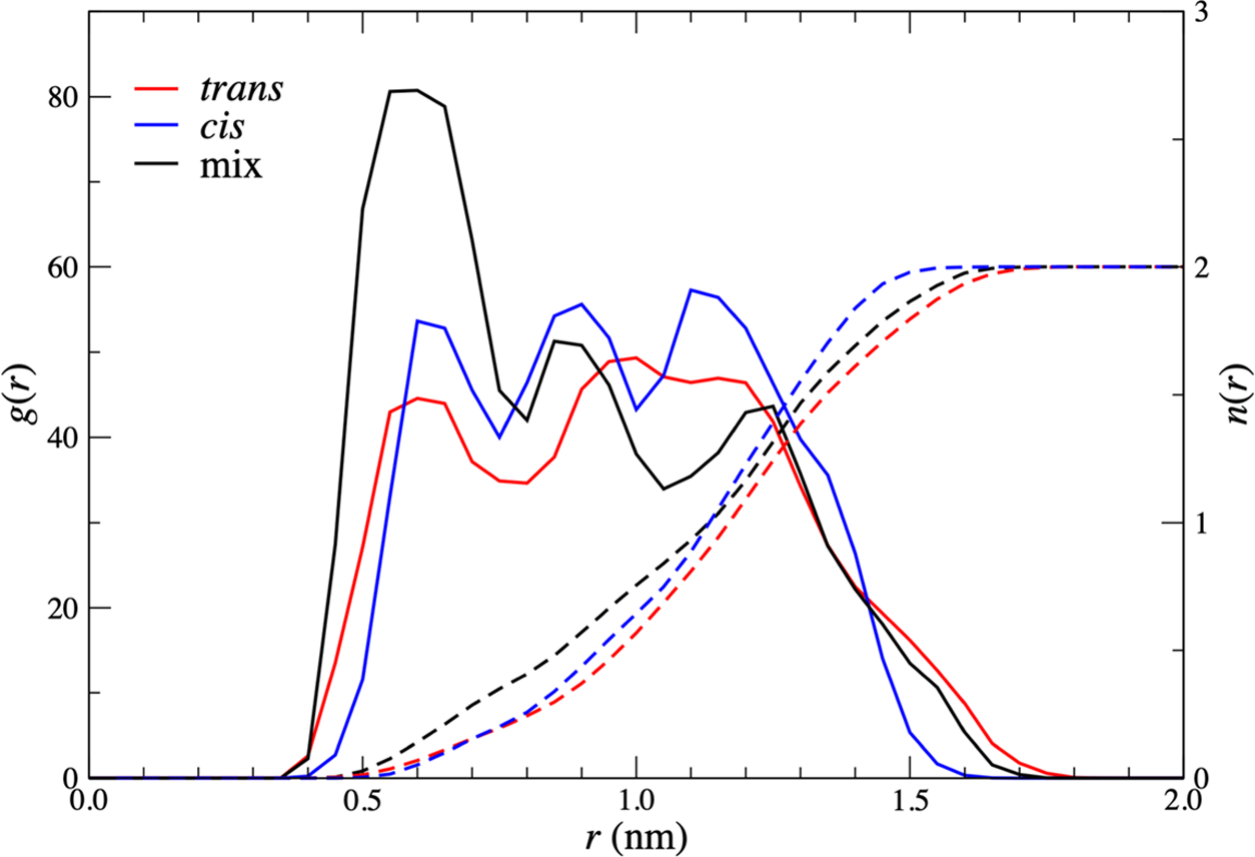
Radial distribution functions, *g*(*r*), (solid lines) and cumulative coordination numbers, *n*(*r*), (dashed lines) for a bound nitrate of one complex, with the bound TBP phosphate of the other. Data are shown for a cis–cis dimer (blue), a cis–trans dimer (black), and a trans–trans dimer (red). The Zr–Zr distance is 0.95 nm, corresponding to the position of the global minimum of the cis–cis PMF.

Figure 10 shows, again, for all three dimers at a separation of 0.95 nm, the radial distribution between a nitrate on one complex and a bound TBP phosphate on the other. The distance is that between the nitrogen atom on the nitrate and the TBP phosphorous atoms. Figure 10 also shows the corresponding cumulative coordination numbers. At large distances, this quantity tends to two. Each radial distribution function has a peak at a relatively short distance of 0.5 nm or thereabouts. The coordination number plots indicate approximately 0.5–0.75 phosphates within a distance of 0.95 nm. This provides evidence for direct nitrate–phosphate interactions, but again the coordination numbers are similar for all three dimers.

**Figure 11:**
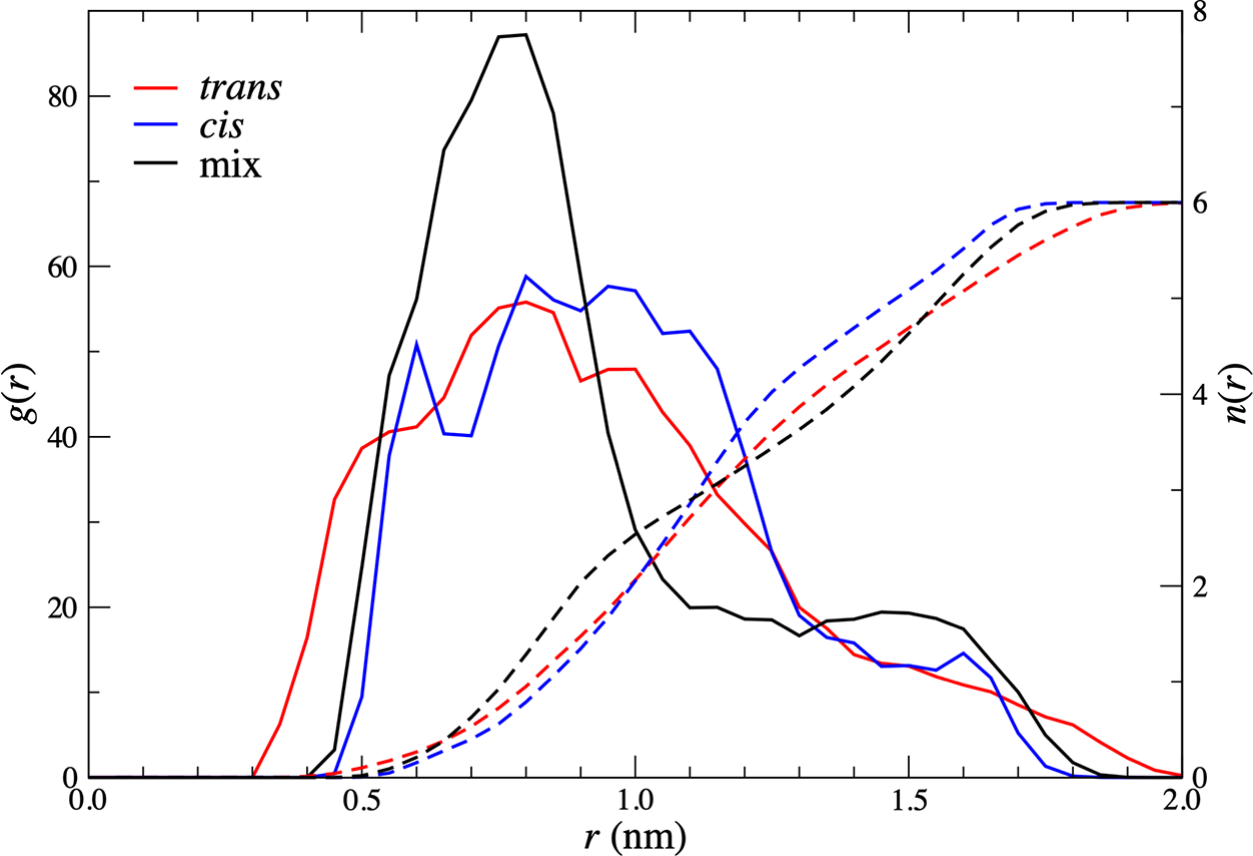
Radial distribution functions, *g*(*r*), (solid lines) and cumulative coordination numbers, *n*(*r*), (dashed lines) for a bound phosphate of one complex with the bound TBP carbon-connecting oxygens of the other. Data are shown for a cis–cis dimer (blue), a cis–trans dimer (black), and a trans–trans dimer (red). The Zr–Zr distance is 0.95 nm, corresponding to the position of the global minimum of the cis–cis PMF.

Finally, we consider the distribution of the TBP oxygens in one complex around the phosphorous atom of the second. Here, there are two distinct types of oxygen atoms. One is the phosphoryl oxygen attached to the Zr atom. The other oxygen connects the P atom to the hydrocarbon chain. The curves for the phosphoryl oxygen are shown in Supporting Information, Figure S7, and again they are very similar for all three dimers. The curves for the other carbon-connecting oxygen atoms are shown in Figure 11. Clearly, there are short-range interactions in evidence, and yet again, the differences between the three types of dimer are rather small. None of this analysis gives any indication as to why the potentials of mean force, at the cis–cis PMF minimum, should be most negative for cis–cis and least negative for trans–trans.

We next turn to the role of electrostatic interactions. The average electrostatic energies between the two complexes at the cis–cis PMF minimum, omitting the long-range contributions to the Ewald sum, were −20.8 ± 1.1 kJ mol^–1^ for cis–cis, −16.4 ± 0.7 kJ mol^–1^ for cis–trans, and −12.8 ± 0.7 kJ mol^–1^ for trans–trans. The corresponding van der Waals or atom–atom Lennard-Jones interactions between these complexes at this separation were −36.5 ± 1.4 kJ mol^–1^ for cis–cis, −39.1 ± 1.1 kJ mol^–1^ for cis–trans, and −38.3 ± 1.5 kJ mol^–1^ for trans–trans. The van der Waals interactions are rather similar, but the ordering of the electrostatic energies follows the same trend as the PMFs at this separation, suggesting that electrostatic interactions play an important role in determining the relative stabilities of these dimers.

The position of the trans–trans PMF minimum is, however, 0.85 nm. At this separation, the energies of the trans dimer are 1.7 ± 5.1 kJ mol^–1^ (electrostatic) and −61.6 ± 2.0 kJ mol^–1^ (van der Waals). By decreasing the separation, the trans dimer has sacrificed electrostatic energy to obtain a considerably more favorable van der Waals interaction. Figure S9 indicates an increased number of P–O interactions at the PMF minimum compared to that at the larger 0.95 nm separation.

It is interesting to explore to what extent dipole–dipole interactions can account for these trends. As noted previously, the DFT calculations on the isolated trans and cis complexes gave dipole moments of 6.453 and 15.224 D, respectively. The DFT dipoles were obtained from the electron density of the complex evaluated at the minimum energy geometry. The force field, however, makes use of partial charges, and the complex is also flexible. Our estimates of the average dipoles of the isolated complexes in solution are 13.25 D for cis and 9.35 D for trans. The simulation setup was the same as that described earlier for the PMF calculations, and the Gromacs gmx dipoles tool was used for this analysis. We then calculated the total dipole of the dimer at its equilibrium distance, as well as the dipole moments of the two individual complexes, and thereby calculated cos θ_12_ = ⟨μ_1_·μ_2_⟩/(⟨μ_1_⟩⟨μ_2_⟩), where μ_1_ and μ_2_ are the two dipoles. We obtained values for cos θ_12_ of −0.47, −0.21, and −0.43 for cis–cis, cis–trans, and trans–trans, respectively. The corresponding angles are 120, 100, and 115°, respectively, indicating the dipoles are not aligned head-to-tail as would be expected for pure dipole–dipole interactions. If this result is combined with the atom–atom correlations previously discussed and visual inspection of the complexes, the general picture is that the complexes are arranged so as to allow interaction between the TBP ligand, and the nitrates are tilted to reduce nitrate–nitrate interactions but not to the same extent as a dipole–dipole analysis would predict.

**Figure 12:**
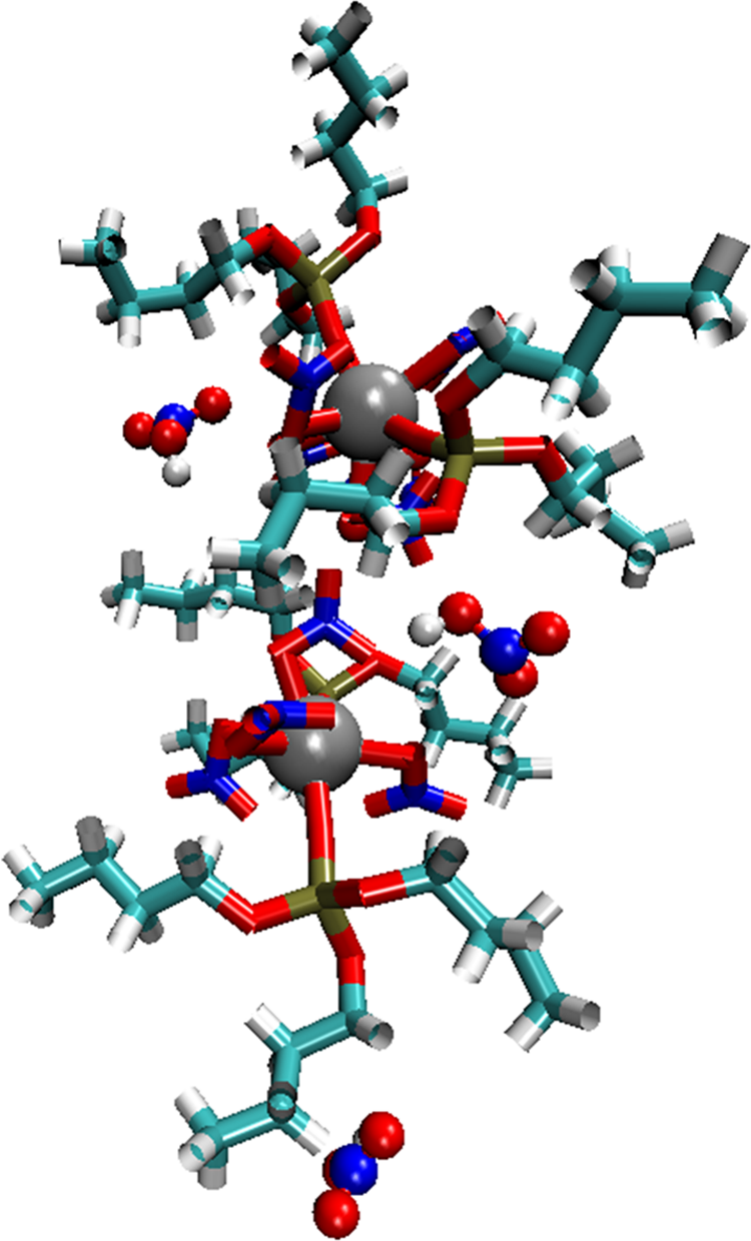
Snapshot of the trans–trans dimer at its equilibrium separation showing a possibly bridging nitric acid molecule. Zr, P, O, N, C, and H atoms are colored gray, gold, red, blue, cyan, and white, respectively.

We finally consider briefly the trans–trans dimer and the positions of its minimum. As the snapshot in Figure 12 indicates, a nitric acid molecule is often observed between the two complexes, albeit to one side of the Zr–Zr line of center. As noted earlier, the radial distribution function for nitric acid around a Zr atom is very similar in the dimer to that of an isolated complex, so the potentially bridging nitric acid is not unusually close to either Zr atom. If the nitric acid does indeed link the two complexes, it is far from clear how the linkage works. It is thus a possibility that a nitric acid bridge is involved in stabilizing the trans–trans dimer, but this is far from certain.

In summary, it is our belief that electrostatic interactions play a key role in determining the relative stabilities of the three types of dimer, but it is almost certain that there is more to it than this. We note, for example, that the atomic partial charges are not identical in the cis and trans isomers and that solvent effects will also be at play. Finally, we confess that we have no clear explanation as to why there are two special distances in the PMFs. To unravel this would require a much deeper analysis.

Finally, this discussion has focused on structure and energy but has not considered entropic effects. It has not proved possible to extract enthalpies and entropies from the PMF simulations. Unfortunately, the errors are too great to obtain meaningful results. We have, however, estimated an enthalpy and entropy of dimerization for a cis system at 298 K from a series of simulations at various temperatures. Details are given in the following section, but we have calculated the number of monomers and dimers from simulation at five different temperatures and thereby obtained the equilibrium constants for dimerization at these temperatures. As Figure S10 indicates, this yielded Δ_dim_*G* = −9.9 kJ mol^–1^, Δ_dim_*H* = −18.7 kJ mol^–1^, and −*T*Δ_dim_*S* = +8.8 kJ mol^–1^. There is thus a favorable enthalpy change but an unfavorable entropic contribution. The enthalpy term is dominant, but the entropic contribution is significant. A similar trend is found from analyzing the Boltzmann weighted average dipole–dipole or Keesom interaction. Here, the entropic contribution is related to a reduction in the orientational freedom of the dipoles as they approach one another, and it is possible that such effects are also playing a role in our systems of complexes.

### Aggregation Model

In light of the calculated PMFs, we may construct an isodesmic aggregation model somewhat along the lines of that proposed by Akinshina et al.^57^ The analysis is based on Wertheim’s thermodynamic perturbation theory (TPT).^58−61^ In this approach, a cluster is not defined in terms of a distance criterion. Instead, the underlying statistical mechanical approach is to divide the Mayer function into a repulsive contribution, including only the repulsive part of the pair potential and an attractive contribution. The density of a cluster is then defined in terms of graphs that contain at least one attractive bond between the associated species. Thus, in the absence of attractions, there is no association. To apply this methodology here, the PMF, denoted by *W*(*r*), is decomposed into a repulsive, *W*_rep_, and an attractive, *W*_att_, contribution based on the Weeks–Chandler–Andersen split,^62^ viz.
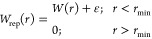
1
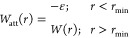
2Here, ε is the well depth of the PMF and *r*_min_ is the position of the minimum. An effective equilibrium constant, Δ_αβ_, is then given by
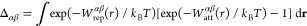
3where *k*_B_ Boltzmann’s constant and *T* is the temperature. α and β take on the labels cis and trans. This corresponds to Wertheim’s expression in the limit of a low density of aggregating particles. Using this result, our PMF data give Δ_trans,trans_ = 74.9 nm^–3^, Δ_trans,cis_ = 131 nm^–3^, and Δ_cis,cis_ = 523 nm^–3^. We may convert these values to standard equilibrium constants based on an ideal molar reference state, following Akinshina et al.^57^ We then obtain *K*_trans,trans_ = 45.1, *K*_trans,cis_ = 78.9, and *K*_cis,cis_ = 315. The corresponding standard Gibbs energies of dimerization are Δ_dim_*G*_trans,trans_^0^ = −9.44 kJ mol^–1^, Δ_dim_*G*_trans,cis_^0^ = −10.8 kJ mol^–1^, and Δ_dim_*G*_cis,cis_^0^ = −14.3 kJ mol^–1^. These Gibbs energies differ from the well depths of the PMFs reported earlier as the shape of the potential well, in addition to its minimum value, plays a role in determining the equilibrium constant for aggregation. It is far from straightforward to give a rigorous estimate of the errors on these quantities, but it is reasonable to suppose that the Gibbs energy errors are similar in magnitude to those given for the PMF well depths.

If one wished to retain a distance criterion for defining a cluster, as is normal in simulation studies, then one may follow Chialvo et al.^63−65^ and calculate
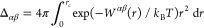
4where *r*_c_ is the distance criterion for the cluster and the integral involves the full PMF. If we set *r*_c_ =1.25 nm, as was our choice in our simulations, then we obtain *K*_trans,trans_ = 30.9, *K*_trans,cis_ = 72.9 and *K*_cis,cis_ = 300. The corresponding standard Gibbs energies of dimerization are Δ_dim_*G*_trans,trans_^0^ = −8.50 kJ mol^–1^, Δ_dim_*G*_trans,cis_^0^ = −10.6 kJ mol^–1^, and Δ_dim_*G*_cis,cis_^0^ = −14.1 kJ mol^–1^. The two sets of values are very similar.

There are pros and cons for each approach. The Wertheim method does not require a cutoff distance, which can be somewhat arbitrary, and it retains the physical idea that a harshly repulsive interaction potential cannot produce clusters. On the other hand, unlike the Chialvo method, one cannot directly compare its predictions against simulation.

### Thermodynamics of Aggregation

**Figure 13:**
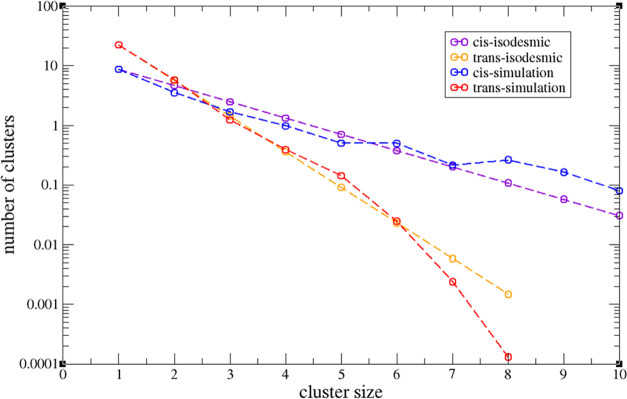
Semi-logarithmic plots of the cluster size distributions for the pure cis (blue) and the pure trans (red) systems. Comparison is made with the isodesmic predictions—cis (purple) and trans (yellow). In these plots, the isodesmic plots are straight lines.

We now turn to the cluster size distributions as obtained from simulations to extract thermodynamic information about cluster formation. As noted earlier, simulation makes use of a distance criterion to define a cluster, while Wertheim’s association theory employs a definition based on the form of the PMF. For strong interactions, the calculated cluster distribution will not be expected to depend strongly on the distance criterion used, but for weak interactions, this dependence is likely to be stronger. As noted previously, we use a distance criterion of a distance of 1.25 nm for all Zr complexes. With these caveats in mind, we first consider the all-trans system of 40 complexes, where the average volume was 2201.6 nm^3^. We calculate equilibrium constants based on the molar concentrations of the species, and we assume we are in Henry’s law limit, so we can ignore activity coefficients. The volume fraction of the complexes in this system is 0.019, so there is justification for this approximation, but the extent to which we may neglect interactions between clusters does warrant future study. The equilibrium constant for forming a trans dimer from two trans monomers, *K*_tt_, based on the simulation values for the concentrations of monomers and dimers, is 15.4, while the equilibrium constant for forming a trimer from a dimer and a monomer, *K*_t:tt_, is 12.6. The isodesmic approximation assumes that the equilibrium constant for forming a cluster of aggregation number *n* from a monomer and an (*n* – 1) cluster is independent of *n*. A fit to the simulation data then gives an equilibrium constant of *K*_tt_^iso^ = 15.3. The predicted isodesmic distribution is compared with simulation in Figure 13 and shows reasonable agreement. We note that very few clusters with aggregation numbers greater than 6 are observed in the simulation, so there is high statistical uncertainty on these points. Also, it is likely that the distribution of large aggregates is highly sensitive to finite system size effects. The isodesmic equilibrium constant corresponds to a Gibbs energy change of – 6.8 kJ mol^–1^ at the simulation temperature of 298 K.

If this procedure is repeated for the pure cis system, where there are again 40 complexes and where the average volume is 2291.7 nm^3^, the dimer equilibrium constant, *K*_cc_, is 64.9, the trimer equilibrium constant, *K*_c;cc_, is 75.7, and the isodesmic equilibrium constant, *K*_cc_^iso^, is 91.4. The latter corresponds to a Gibbs energy change of −11.2 kJ mol^–1^. The predictions of the isodesmic approximation are again compared with simulation in Figure 13. The caveats noted above about large cluster numbers again apply but, all in all, the isodemic approximation is again reasonable. Within error, the Gibbs energies of dimerization calculated here are similar to those obtained via the potential of the mean force (PMF) route.

Before moving on to the mixed cis/trans system, it is worth noting that sometimes the isodesmic model may be improved by assuming a single equilibrium constant for the formation of trimers and higher order clusters but to allow the dimer equilibrium constant to have a different value.^66^ This does lead to a small improvement in the predictions, but given the inevitable statistical errors in the simulation data, the system size restrictions, and the sensitivity to the cluster distance criterion, we believe we are not yet at a stage to significantly improve the model beyond the basic isodesmic approach.

We now turn to the cis/trans mixture, where the average volume is 2246 nm^3^ and where there are 20 cis isomers and 20 trans isomers. We again note that the simulation force field does not allow for chemical equilibrium between cis and trans isomers, and we use the 1.25 nm cluster distance criterion. The data in Table 1 indicate that as the aggregates increase in size, the proportion of cis isomers within them increases.

We have made attempts to calculate the average number of clusters as a function of both trans and cis numbers, but the results are erratic. The statistical errors are large due to the small number of clusters observed, and we find a high sensitivity to the cluster distance criterion. Further simulation work will be needed for a quantitative analysis, but what is clear from our current results is that it is the cis isomer that preferentially forms aggregates. In the experimental system, our hypothesis is that the cis and trans isomers spontaneously interconvert. According to our quantum mechanical calculations, the Gibbs energy of converting an isolated cis isomer to a trans isomer in an implicit solvent is 5.8 kJ mol^–1^, i.e., the trans-state is slightly favored. This corresponds to an equilibrium constant of 0.1, so thermodynamically, this conversion is relatively easy. The question then arises as to whether there exists an accessible pathway. While we cannot answer this definitively, we note that NMR studies on related systems indicate a significant rate of exchange of ligands on the NMR time scale.^67^ Given this, we may reasonably assume that cis–trans interconversion is also likely to be happening on this time scale.

In light of all of the system information, our hypothesis is that at low metal concentrations, where the Zr complexes mainly exist as monomers, the trans/cis ratio is approximately 10:1. As the concentration increases, clustering occurs. The Gibbs energy is lowered due to the favorable Gibbs energies of aggregation. As, however, the Gibbs energy of clustering is more negative for cis than trans isomers, the system’s Gibbs energy is lowered by the conversion of trans isomers to cis. As the overall metal concentration increases, the proportion of cis isomers also increases. It is our hope that future spectroscopic studies on this system will be able to show that the ratio of cis to trans isomers will increase with increased overall zirconium concentration.

## Conclusions

We have studied a phase consisting of Zr(NO_3_)_4_(TBP)_2_ complexes incorporated within a mixture of water, nitric acid, TBP, and *n*-octane, with the composition corresponding to that of sample 5, studied by Motokawa et al.^21^ An analysis of the SANS profiles indicated a hierarchical aggregation structure, with the zirconium complexes first aggregating into clusters and then these clusters aggregating into superclusters. This clustering was also predicted by molecular dynamics simulation, but on improving the force field, we discovered that the trans clusters previously studied showed little tendency to aggregate. Our quantum mechanical calculations indicate, however, that an isolated trans isomer is only slightly more stable, by ca. 5.8 kJ mol^–1^ than the cis isomer. Our current studies indicate that cis isomers aggregate readily. Our hypothesis is therefore that there is a trans–cis chemical equilibrium and that, with increased zirconium loading, the clustering drives this equilibrium in favor of the cis isomer.

The striking difference between the cis and trans isomers is the presence of a polar patch on the cis isomer. Our simulations indicate that electrostatic interactions play an important role in determining the relative stabilities of the dimers. The cis–cis dimer is the most strongly bound, followed by the cis–trans and then the trans–trans dimers. This is consistent with the observation from simulation of a mixed cis/trans system that the cis isomers were predominantly in the larger aggregates.

For a more complete analysis, we will need a simulation methodology that allows the Zr complex isomers to spontaneously interconvert and, in addition, we will require a force field that permits the ionic dissociation of nitric acid, for protonation effects may also play a role. What we hope is clear, though, is that isomerism has a very significant effect on clustering. It is likely that these effects are not restricted to zirconium/TBP complexes but may also apply to many other metal systems. We believe that this is a topic worthy of future investigation and that a consideration of such isomeric equilibrium may help improve the quality of flow sheet modeling for extraction processes and help avoid third phase formation.

## Supporting Information Available

Structures and partial charges for the zirconium complexes (Section S1); simulated cluster distributions—further details (Section S2); pair correlation functions and coordination numbers for Zr dimers (Section S3); temperature effects of dimerization for the cis system (Section S4) (PDF)
GROMACS input files for the study of the 40 complex cis, trans and mixed systems (ZIP)
