# Supplementary material for: Hierarchical Aggregation in a Complex Fluid—The Role of Isomeric Interconversion
Source: J Phys Chem B. 2023 Feb 23;127(9):2052–65. doi: 10.1021/acs.jpcb.2c07527 (PMC10009746; doi:10.1021/acs.jpcb.2c07527)
Supplement: Supplementary file 1 — jp2c07527_si_001.pdf [file jp2c07527_si_001.pdf]

Hierarchical Aggregation in a Complex Fluid – the role of isomeric inter-conversion

Daniel Massey,† Christopher D. Williams,† Junju Mu,†,‡ Andrew J. Masters,†,*

Ryuhei Motokawa,#,* Noboru Aoyagi,§ Yuki Ueda,# and Mark R. Antonio||

†*Department of Chemical Engineering, School of Engineering, The University of Manchester, Oxford Road, Manchester M13 9PL, United Kingdom*

‡ *Dalian Institute of Chemical Physics, CAS 457 Zhongshan Road, Dalian, China*

#*Materials Sciences Research Center, Japan Atomic Energy Agency, Tokai, Ibaraki 319-1195, Japan*

§*Advanced Science Research Center, Japan Atomic Energy Agency, Tokai, Ibaraki 319-1195, Japan*

||*Department of Chemistry, Colorado School of Mines, Golden, CO 80401, United States.*

Supporting Information

1. Structures and partial charges for the zirconium complexes

a) Atomic co-ordinates (Å)

Trans **- complex**

Zr	-0.08534	-0.51391	0.70213
P	3.48472	-0.21150	-0.30379
O	3.71433	1.01617	-1.24268
O	3.99398	-1.42849	-1.13477
O	4.50341	-0.02721	0.86390
O	2.08396	-0.34890	0.18438
C	3.08740	1.14254	-2.55776
C	3.31218	2.55485	-3.05457
C	2.71209	2.76813	-4.44681
C	2.92656	4.18933	-4.96654
C	3.78388	-2.83567	-0.79277
C	5.05092	-3.43142	-0.20738
C	4.89814	-4.91716	0.14790
C	4.71354	-5.84447	-1.05564
C	4.25646	0.78595	2.05635
C	4.93477	2.13675	1.94104
C	4.75398	2.96490	3.21671
C	5.43077	4.33242	3.12976

H	3.55187	0.40537	-3.21255
H	2.02654	0.91982	-2.45745
H	2.86468	3.25502	-2.34569
H	4.38575	2.75889	-3.07643
H	3.15278	2.05303	-5.14765
H	1.64113	2.54880	-4.41725
H	2.49108	4.31729	-5.95819
H	3.98978	4.42671	-5.03734
H	2.46669	4.92430	-4.30336
H	3.50457	-3.30367	-1.73309
H	2.94374	-2.91316	-0.10509
H	5.31784	-2.87033	0.69005
H	5.86858	-3.30080	-0.92114
H	4.06080	-5.03934	0.84050
H	5.79054	-5.22207	0.69855
H	4.68829	-6.88781	-0.73923
H	5.53534	-5.73388	-1.76646
H	3.78337	-5.64757	-1.59024
H	4.66671	0.20053	2.87672
H	3.18170	0.87858	2.20099
H	4.52205	2.67888	1.08718
H	5.99877	1.98634	1.74129
H	5.15762	2.41148	4.06960
H	3.68693	3.09786	3.41427
H	5.28840	4.90184	4.04886
H	5.02219	4.92196	2.30692
H	6.50520	4.23145	2.96404
P	-3.35199	0.60886	-0.53974
O	-3.62950	2.10423	-0.20298
O	-4.55482	-0.18382	0.05038
O	-3.49454	0.50552	-2.08793
O	-2.03005	0.12328	-0.03953
C	-3.58453	2.65728	1.15307
C	-3.46072	4.16206	1.04915
C	-3.44616	4.82414	2.42994
C	-3.31597	6.34467	2.34871
C	-4.63597	-1.64839	0.07071
C	-5.56186	-2.06022	1.19471
C	-5.73276	-3.58087	1.25551
C	-6.65836	-4.02051	2.38939
C	-2.51268	1.05205	-3.02895
C	-2.97863	0.75303	-4.43749
C	-3.02549	-0.73547	-4.78679
C	-3.45484	-0.98450	-6.23203
H	-4.50782	2.36136	1.65070
H	-2.73958	2.21839	1.68091
H	-2.54260	4.40779	0.51003
H	-4.29444	4.54974	0.45816
H	-4.36266	4.56447	2.96751
H	-2.61941	4.41776	3.01859

H	-4.14698	6.78296	1.79252
H	-2.39111	6.63423	1.84644
H	-3.30842	6.79278	3.34279
H	-3.63387	-2.05032	0.20886
H	-5.01662	-1.95743	-0.90248
H	-6.53377	-1.58065	1.05247
H	-5.15593	-1.69505	2.14081
H	-4.75367	-4.05051	1.38133
H	-6.12730	-3.94196	0.30120
H	-7.65562	-3.59115	2.27429
H	-6.27009	-3.70325	3.35882
H	-6.76430	-5.10558	2.41276
H	-1.54938	0.59275	-2.81268
H	-2.44616	2.12461	-2.85141
H	-2.28667	1.26865	-5.11088
H	-3.95981	1.20999	-4.59270
H	-3.71327	-1.24488	-4.10886
H	-2.04078	-1.17753	-4.61274
H	-4.45107	-0.58050	-6.42413
H	-3.48106	-2.05111	-6.45729
H	-2.76612	-0.51359	-6.93672
N	-0.59488	-2.23592	-1.47342
O	-1.12658	-2.46065	-0.35173
O	-0.77229	-2.90900	-2.45657
O	0.16918	-1.19818	-1.48055
N	-1.86442	-0.71742	2.86777
O	-2.65261	-0.85238	3.76771
O	-1.51207	-1.68559	2.10460
O	-1.31112	0.38424	2.57222
N	1.42083	-2.07374	2.40598
O	2.11376	-2.73142	3.13274
O	0.95800	-2.50597	1.29724
O	1.08011	-0.86806	2.66480
N	0.55703	2.17389	0.18817
O	0.54113	1.62209	1.34375
O	0.87094	3.32600	0.02207
O	0.21253	1.39693	-0.75272

***Cis*- complex**

Zr	0.79616	0.83297	1.47029
P	-2.52651	-0.05832	0.27301
O	-2.99324	1.14280	-0.60228
O	-3.65496	-0.21330	1.34600
O	-2.53928	-1.27904	-0.70526
O	-1.16649	0.03977	0.87027
C	-3.28775	2.47300	-0.05714
C	-3.49154	3.42537	-1.21431
C	-3.83817	4.83523	-0.72690

C	-4.04418	5.81800	-1.87898
C	-3.51370	-0.83574	2.66376
C	-3.76421	-2.33143	2.59760
C	-3.66263	-3.00970	3.97174
C	-4.75914	-2.60900	4.96118
C	-3.71320	-1.67271	-1.47533
C	-3.40052	-2.96950	-2.19210
C	-4.58112	-3.45004	-3.04027
C	-4.28467	-4.75891	-3.77155
H	-4.18281	2.37986	0.55726
H	-2.44723	2.77779	0.56225
H	-2.57704	3.45591	-1.80944
H	-4.29153	3.04591	-1.85586
H	-4.74231	4.79829	-0.11198
H	-3.03819	5.19952	-0.07708
H	-4.28810	6.81403	-1.50811
H	-4.85912	5.49715	-2.53116
H	-3.14313	5.90171	-2.48894
H	-4.25342	-0.32374	3.27285
H	-2.52173	-0.60831	3.04727
H	-3.03281	-2.78261	1.92307
H	-4.75326	-2.51089	2.16697
H	-2.67950	-2.80444	4.40300
H	-3.70239	-4.08962	3.81435
H	-4.67280	-3.18227	5.88471
H	-5.75200	-2.79702	4.54659
H	-4.70636	-1.55371	5.23176
H	-3.93906	-0.86985	-2.17667
H	-4.55260	-1.78919	-0.78903
H	-3.13777	-3.73083	-1.45360
H	-2.52188	-2.82307	-2.82496
H	-4.84448	-2.67736	-3.76822
H	-5.45910	-3.58067	-2.40112
H	-5.13915	-5.07914	-4.36831
H	-4.05197	-5.55999	-3.06752
H	-3.43154	-4.65048	-4.44384
P	2.02150	-0.66486	-1.64085
O	1.53265	0.03757	-2.94507
O	1.69253	-2.17798	-1.84578
O	3.57279	-0.58294	-1.68882
O	1.39792	-0.10441	-0.40475
C	0.13346	0.29562	-3.26875
C	0.08914	1.21549	-4.47065
C	-1.34713	1.49938	-4.91787
C	-1.40968	2.43376	-6.12577
C	2.01293	-3.21214	-0.86342
C	1.39070	-4.51300	-1.32441
C	1.70111	-5.66186	-0.36085
C	1.07893	-6.98530	-0.80471
C	4.35828	0.65852	-1.64169

C	5.81726	0.30442	-1.82962
C	6.41620	-0.53415	-0.69886
C	7.89977	-0.83200	-0.91220
H	-0.33877	-0.66450	-3.47934
H	-0.34543	0.74674	-2.40096
H	0.59068	2.15179	-4.21690
H	0.65045	0.75771	-5.28911
H	-1.84672	0.55645	-5.16028
H	-1.90492	1.93998	-4.08749
H	-0.88530	2.00644	-6.98270
H	-0.94803	3.39674	-5.90106
H	-2.44090	2.62146	-6.42699
H	1.62618	-2.89938	0.10474
H	3.09908	-3.28779	-0.80699
H	1.76250	-4.75368	-2.32363
H	0.30920	-4.37993	-1.40854
H	1.34060	-5.40550	0.63902
H	2.78493	-5.78004	-0.27416
H	1.44672	-7.28431	-1.78821
H	-0.00837	-6.90869	-0.86696
H	1.31534	-7.78664	-0.10400
H	4.17313	1.13084	-0.68089
H	3.99694	1.30484	-2.43917
H	6.35820	1.25250	-1.91033
H	5.94275	-0.20652	-2.78841
H	5.86399	-1.47235	-0.61186
H	6.27843	-0.00726	0.24906
H	8.06299	-1.38517	-1.83967
H	8.30462	-1.42940	-0.09454
H	8.48364	0.08899	-0.97017
N	0.46371	-1.46998	2.98799
O	-0.13446	-0.38746	3.27136
O	0.33077	-2.49023	3.60871
O	1.22501	-1.37697	1.95642
N	0.23123	3.21185	2.73816
O	-0.06462	4.23864	3.27952
O	-0.55317	2.19095	2.70014
O	1.33703	3.01713	2.14039
N	3.24624	0.90253	2.74485
O	4.32729	0.95356	3.25726
O	3.07308	0.90816	1.47046
O	2.15305	0.83012	3.39350
N	0.80572	2.95645	-0.79794
O	-0.01834	2.25788	-0.05174
O	0.31489	3.72033	-1.61346
O	2.01117	2.78328	-0.63602

b) Atomic partial charges (*e*)

Trans - complex

Zr	**4.027**
P	**0.802**
O	**-0.225**
O	**-0.295**
O	**-0.394**
O	**-0.828**
C	**-0.166**
C	**0.045**
C	**0.242**
C	**-0.306**
C	**0.114**
C	**-0.258**
C	**0.027**
C	**-0.142**
C	**0.179**
C	**-0.021**
C	**0.130**
C	**-0.270**
H	**0.118**
H	**0.139**
H	**0.026**
H	**0.002**
H	**-0.032**
H	**-0.042**
H	**0.075**
H	**0.068**
H	**0.063**
H	**0.095**
H	**0.092**
H	**0.117**
H	**0.122**
H	**0.020**
H	**0.029**

H	**0.047**
H	**0.040**
H	**0.031**
H	**0.036**
H	**0.006**
H	**0.036**
H	**0.046**
H	**-0.011**
H	**-0.007**
H	**0.073**
H	**0.062**
H	**0.060**
P	**1.157**
O	**-0.362**
O	**-0.452**
O	**-0.407**
O	**-0.770**
C	**0.022**
C	**-0.022**
C	**0.160**
C	**-0.280**
C	**0.153**
C	**-0.039**
C	**0.170**
C	**-0.284**
C	**0.056**
C	**-0.195**
C	**0.241**
C	**-0.361**
H	**0.076**
H	**0.045**
H	**0.029**
H	**0.048**

H	**-0.017**
H	**-0.010**
H	**0.060**
H	**0.065**
H	**0.073**
H	**-0.010**
H	**0.059**
H	**0.036**
H	**0.045**
H	**-0.009**
H	**-0.024**
H	**0.064**
H	**0.061**
H	**0.074**
H	**0.104**
H	**0.043**
H	**0.087**
H	**0.063**
H	**-0.027**
H	**-0.014**
H	**0.087**
H	**0.085**
H	**0.082**
N	**0.816**
O	**-0.691**
O	**-0.389**
O	**-0.714**
N	**0.854**
O	**-0.412**
O	**-0.647**
O	**-0.790**
N	**0.900**
O	**-0.422**

O	**-0.760**
O	**-0.720**
N	**0.737**
O	**-0.624**
O	**-0.369**
O	**-0.710**

Cis - complex

Zr	**3.809**
P	**1.113**
O	**-0.341**
O	**-0.423**
O	**-0.359**
O	**-0.922**
C	**-0.001**
C	**-0.135**
C	**0.126**
C	**-0.308**
C	**0.228**
C	**-0.246**
C	**0.139**
C	**-0.252**
C	**-0.079**
C	**-0.010**
C	**0.105**
C	**-0.282**
H	**0.103**
H	**0.016**
H	**0.131**
H	**0.080**
H	**0.006**

H	**0.010**
H	**0.079**
H	**0.069**
H	**0.077**
H	**0.067**
H	**0.045**
H	**0.084**
H	**0.103**
H	**-0.004**
H	**0.008**
H	**0.066**
H	**0.061**
H	**0.055**
H	**0.079**
H	**0.097**
H	**0.069**
H	**0.061**
H	**0.012**
H	**0.001**
H	**0.078**
H	**0.064**
H	**0.061**
P	**0.898**
O	**-0.379**
O	**-0.347**
O	**-0.300**
O	**-0.886**
C	**0.327**
C	**-0.236**
C	**0.390**
C	**-0.371**
C	**0.023**
C	**0.033**

C	**0.180**
C	**-0.257**
C	**0.004**
C	**-0.168**
C	**0.272**
C	**-0.338**
H	**0.025**
H	**-0.027**
H	**0.083**
H	**0.051**
H	**-0.049**
H	**-0.119**
H	**0.077**
H	**0.088**
H	**0.081**
H	**0.104**
H	**0.084**
H	**0.028**
H	**-0.010**
H	**-0.025**
H	**-0.030**
H	**0.052**
H	**0.056**
H	**0.067**
H	**0.158**
H	**0.098**
H	**0.062**
H	**0.072**
H	**-0.028**
H	**-0.037**
H	**0.073**
H	**0.077**
H	**0.076**

N	**1.011**
O	**-0.708**
O	**-0.419**
O	**-0.828**
N	**0.782**
O	**-0.365**
O	**-0.686**
O	**-0.655**
N	**0.842**
O	**-0.361**
O	**-0.728**
O	**-0.712**
N	**0.821**
O	**-0.603**
O	**-0.465**
O	**-0.600**

2. The simulated cluster distributions – further details

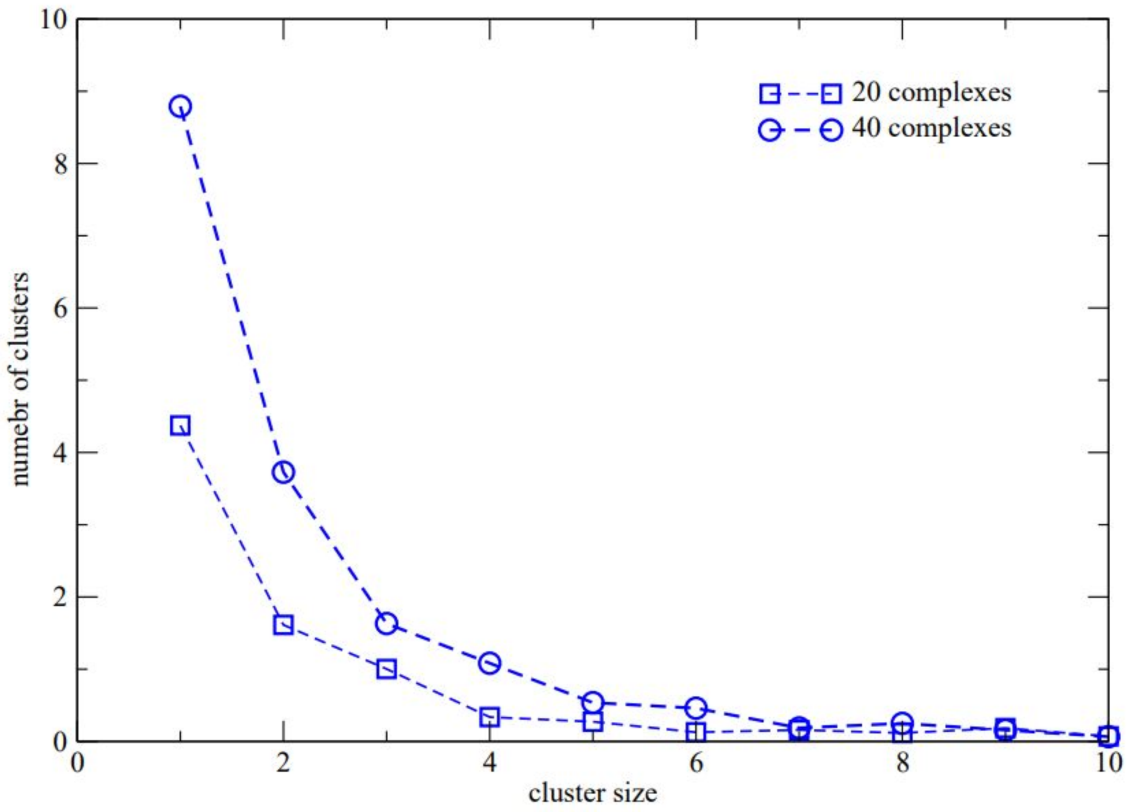

Figure S1. A comparison of the pure *cis* cluster size distributions for simulations with 20 complexes (circles) and 40 complexes (squares)

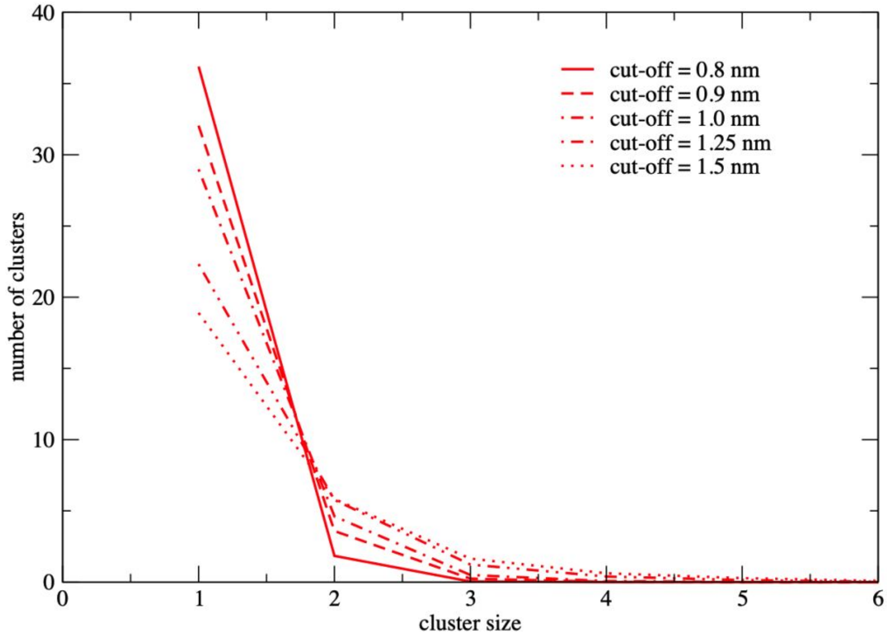

Figure S2. Effect of cluster distance cut-off criterion on the cluster size distribution in the pure *trans* system.

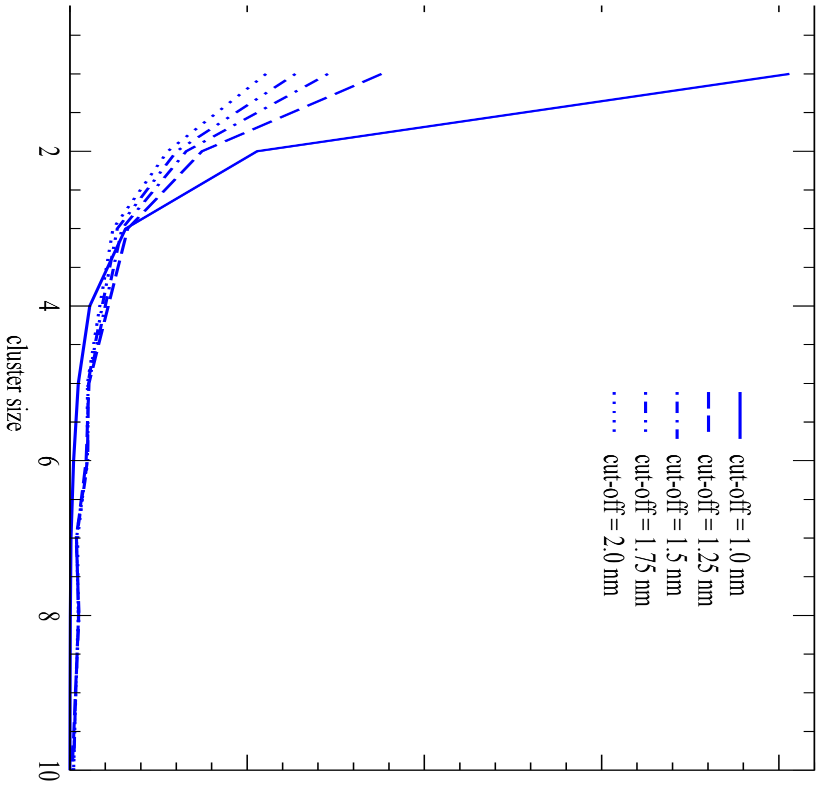

Figure S3. Effect of cluster distance cut-off criterion on the cluster size distribution in the pure *cis* system.

	cut-off = 1.25 nm			cut-off = 1.0 nm		
cluster size	*cis*	*trans*	mix	*cis*	*trans*	mix
1	8.674	22.327	20.354	20.297	28.975	29.445
2	3.540	5.774	4.994	5.280	4.613	4.108
3	1.685	1.227	1.791	1.573	0.495	0.608
4	0.981	0.391	0.604	0.560	0.069	0.100
5	0.503	0.144	0.231	0.236	0.008	0.002
6	0.500	0.025	0.100	0.104	0.000	0.001
7	0.215	0.002	0.015	0.030	0.000	0.000
8	0.265	0.000	0.001	0.013	0.000	0.000
9	0.164	0.000	0.000	0.005	0.000	0.000
10	0.080	0.000	0.000	0.001	0.000	0.000

Table S1. Cluster size distributions in the pure *cis,* pure *trans* and *cis-trans* mixture using cut-offs of 1.25 nm and 1.0 nm.

3. Pair correlation functions and co-ordination numbers for Zr dimers

We present here a set of pair correlation functions and co-ordination numbers for key pairs of atoms. The simulations relate to Zr complex dimers.

a)

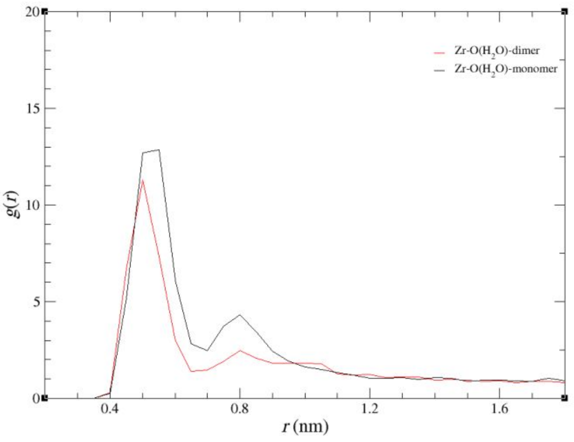

b)

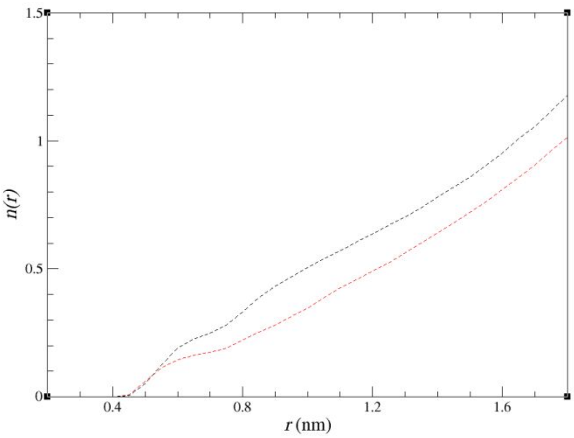

c)

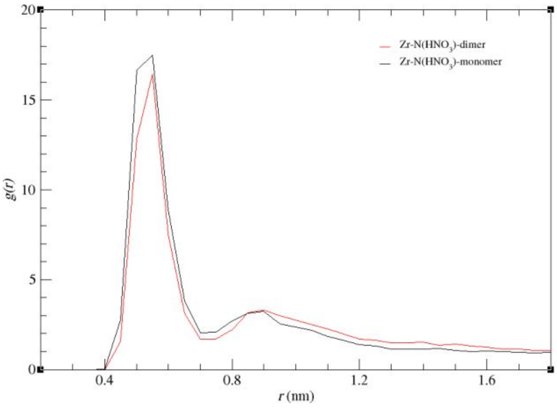

d)

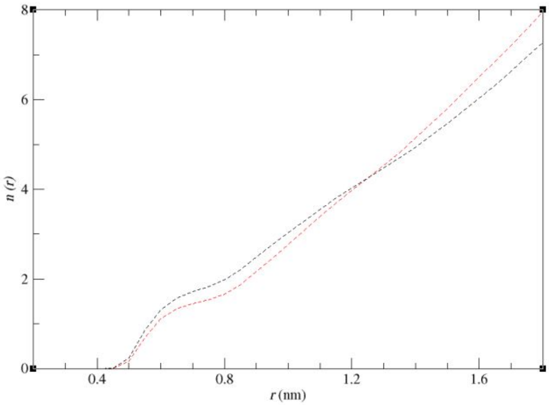

Figure S4. Radial distribution and co-ordination numbers of Zr atoms to O (on H_2O) (radial distribution and co-ordination numbers a and b respectively) and $N(HNO_3)$ (radial distribution and co-ordination numbers c and d respectively) for the *cis-cis* dimer. Data are presented at the PMF minimum (red) and at large Zr – Zr distances (black).

a)

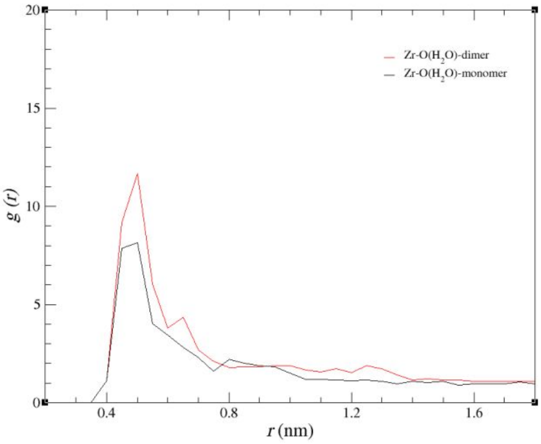

b)

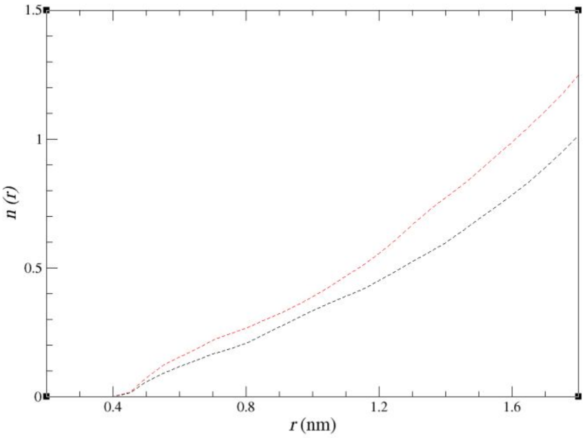

c)

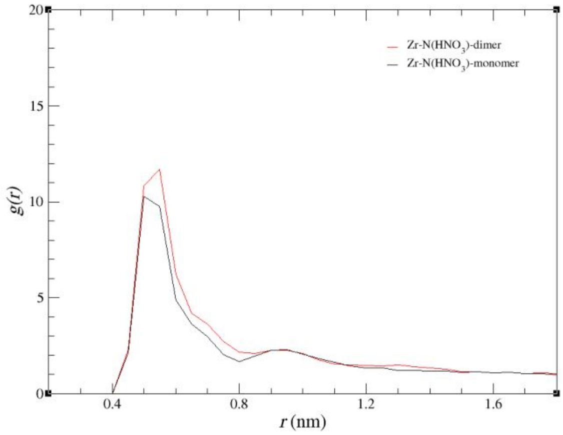

d)

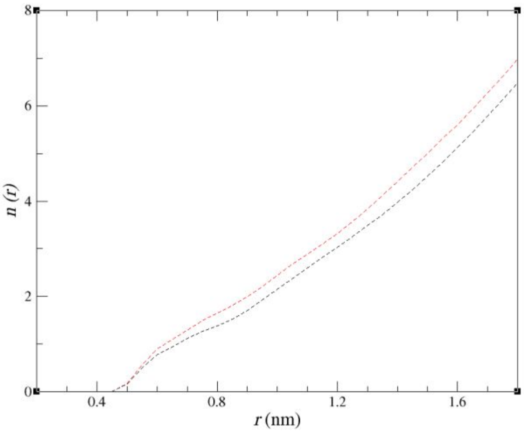

Figure S5. Radial distribution and co-ordination numbers of Zr atoms to O (on H_2O) (radial distribution and co-ordination numbers a and b respectively) and $N(HNO_3)$ (radial distribution and co-ordination numbers c and d respectively) for the *trans-trans* dimer. Data are presented at the PMF minimum (red) and at large Zr – Zr distances (black).

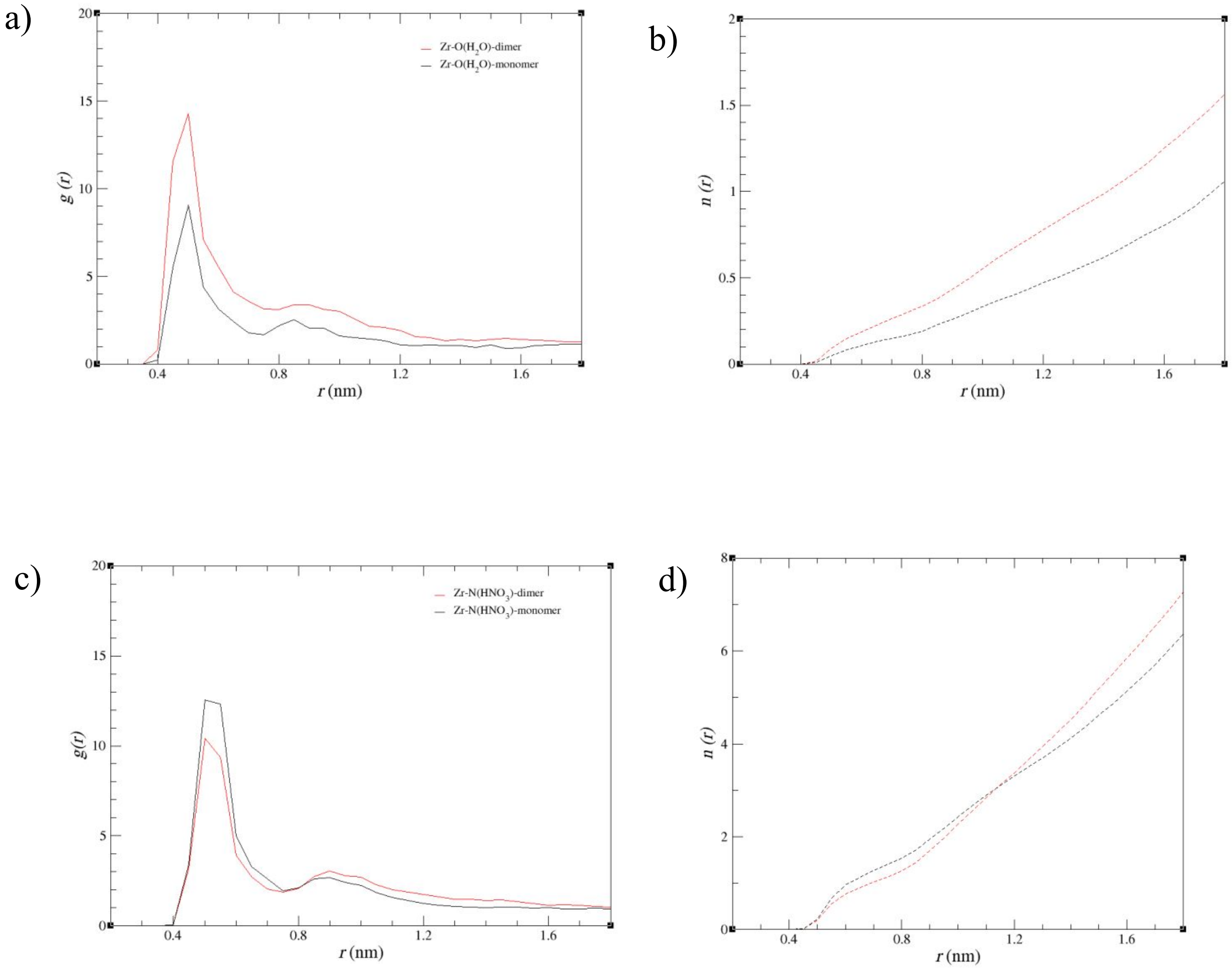

Figure S6. Radial distribution and co-ordination numbers of Zr atoms to O (on H_2O) (radial distribution and co-ordination numbers a and b respectively) and $N(HNO_3)$ (radial distribution and co-ordination numbers c and d respectively) for the *cis-trans* dimer. Data are presented at the PMF minimum (red) and at large Zr – Zr distances (black).

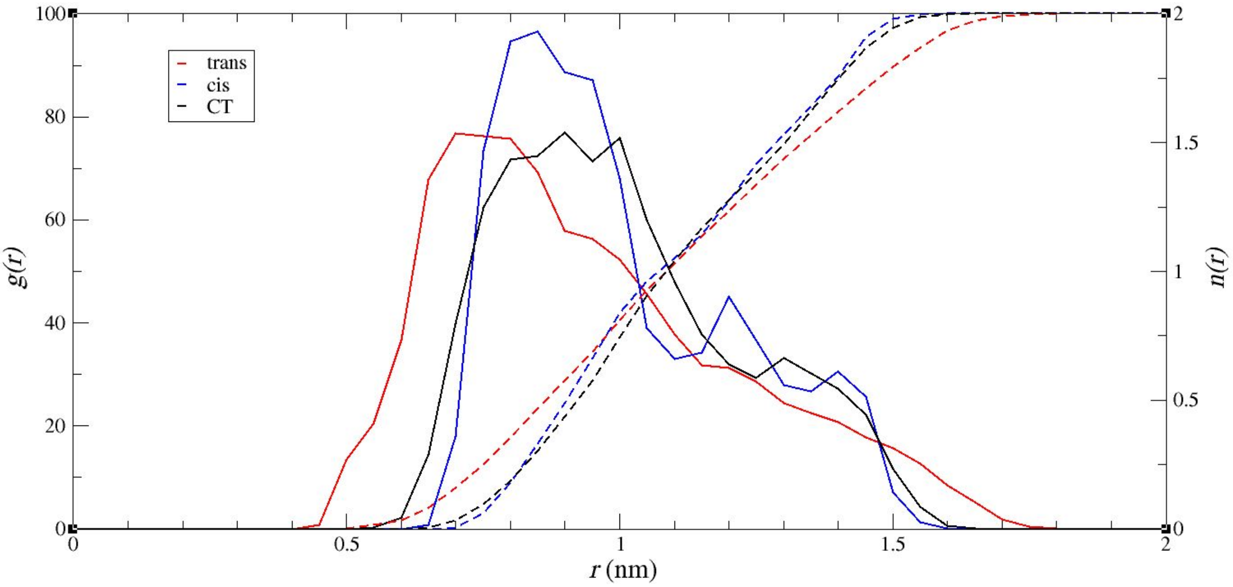

Figure S7. Radial distribution functions, *g(r)*, (solid lines) and cumulative co-ordination numbers, *n(r)*, (dashed lines) for a bound phosphorous atom in one complex to a bound TBP phosphoryl oxygen atom in the other. Data are shown for a *cis-cis* dimer (blue), a *cis-trans* dimer (black) and a *trans-trans* dimer (red). The Zr-Zr distance is 0.95 nm, corresponding to the position of the global minimum of the *cis-cis* PMF.

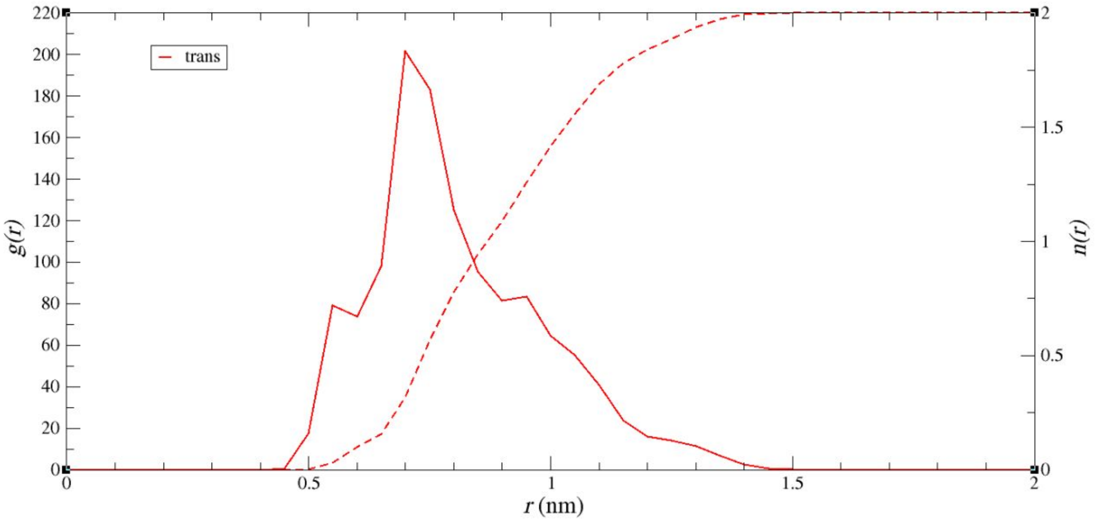

Figure S8. Radial distribution functions, *g(r)*, (solid lines) and cumulative co-ordination numbers, *n(r)*, (dashed lines) for a phosphorous atom in a bound TBP in one complex to a phosphoryl oxygen atom in a bound TBP molecule in the other. Data are shown for a *trans-trans* dimer (red). The Zr-Zr distance is 0.8 nm, corresponding to the position of the global minimum of the *trans-trans* PMF.

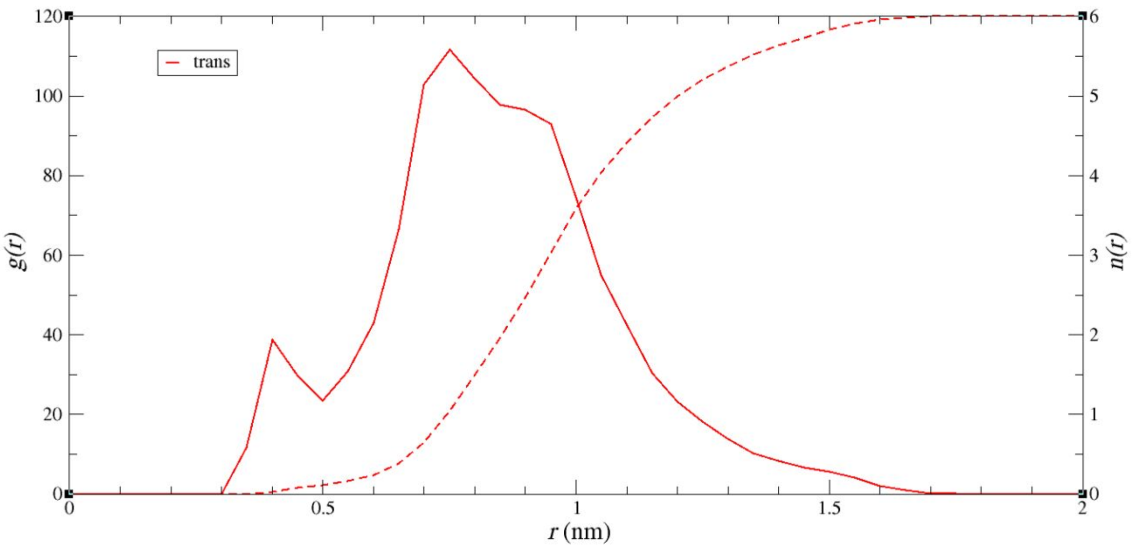

Figure S9. Radial distribution functions, $g(r)$, (solid lines) and cumulative co-ordination numbers, $n(r)$, (dashed lines) for a phosphorous atom in a bound TBP in one complex to a carbon-connecting oxygen atom in a bound TBP molecule in the other. Data are shown for a *trans-trans* dimer (red). The Zr-Zr distance is 0.8 nm, corresponding to the position of the global minimum of the *trans-trans* PMF.

4. Temperature effects of dimerization for the *cis*-system

Simulations were performed for *cis* systems containing 20 complexes at temperatures of 278 K, 288 K, 298 K, 308 K and 318 K. Cluster analysis gave the numbers of monomers and dimers at each temperature, permitting the calculation of the temperature dependent dimerization equilibrium constant, K. A plot of $R\ln K$ vs $1/T$ then permits an estimate of the enthalpies and entropies of dimerization, as shown in Figure **S10**.

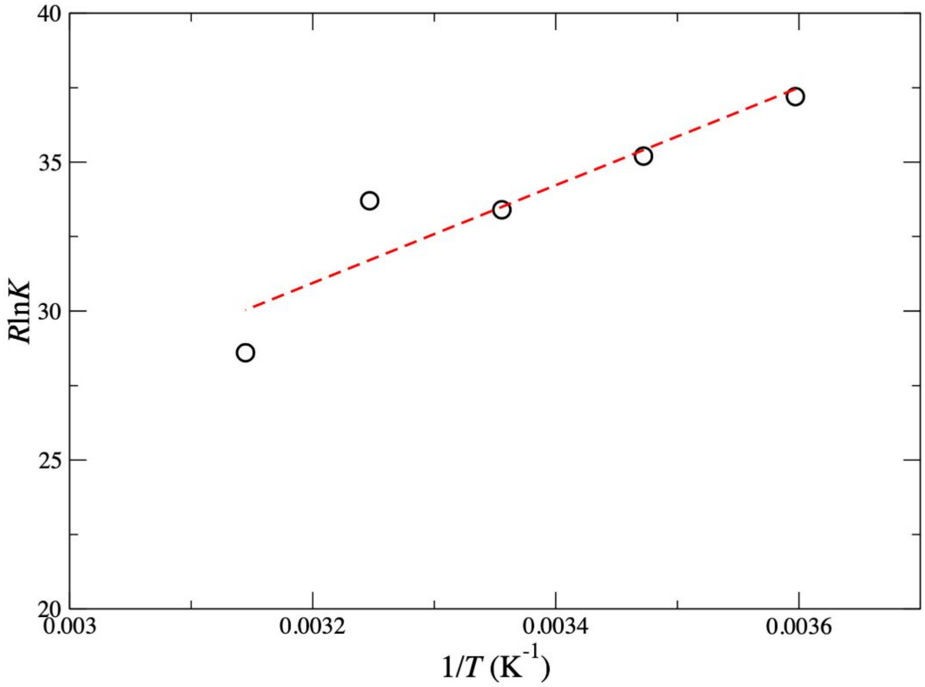

Figure S10. Plot of *R*ln*K* vs 1/*T* for a system of 20 *cis* complexes. Here *K* is the dimerization constant. The slope of the line is the standard enthalpy of dimerization and the intercept is the negative of the standard entropy of dimerization.
